# Glucosamine Downregulates the IL-1β-Induced Expression of Proinflammatory Cytokine Genes in Human Synovial MH7A Cells by *O*-GlcNAc Modification-Dependent and -Independent Mechanisms

**DOI:** 10.1371/journal.pone.0165158

**Published:** 2016-10-24

**Authors:** Akimasa Someya, Takako Ikegami, Koji Sakamoto, Isao Nagaoka

**Affiliations:** 1 Department of Host Defense and Biochemical Research, Juntendo University, Graduate School of Medicine, Bunkyo-Ku, Tokyo 113–8421, Japan; 2 Laboratory of Molecular and Biochemical Research, Research Support Center, Juntendo University, Graduate School of Medicine, Bunkyo-Ku, Tokyo 113–8421, Japan; 3 Koyo Chemical Co., Ltd., Taito-ku, Tokyo110-0005, Japan; Universidad de Jaen, SPAIN

## Abstract

Osteoarthritis (OA) is one of the major joint diseases, and the synovial inflammation is involved in the pathogenesis and progression of OA. Glucosamine (GlcN) is widely used as a dietary supplement for OA, and is expected to exert the antiinflammatory action in OA. However, the detailed mechanism for the antiinflammatory action of GlcN remains poorly understood. In this study, to elucidate the molecular mechanism involved in the GlcN-medicated regulation of synovial cell activation, we comprehensively analyzed the effect of GlcN on the gene expression using a human synovial cell line MH7A by DNA microarray. The results indicated that GlcN significantly downregulates the expression of 187 genes (≤1/1.5-fold) and upregulates the expression of 194 genes (≥1.5-fold) in IL-1β-stimulated MH7A cells. Interestingly, pathway analysis indicated that among the 10 pathways into which the GlcN-regulated genes are categorized, the 4 pathways are immune-related. Furthermore, GlcN suppressed the expression of proinflammatory cytokine genes (such as IL-6, IL-8, IL-24 and TNF-α genes). In addition, GlcN-mediated *O*-GlcNAc modification was involved in the downregulation of TNF-α and IL-8 genes but not IL-6 and IL-24 genes, based on the effects of alloxan, an *O*-GlcNAc transferase inhibitor. Thus, GlcN likely exerts an antiinflammatroy action in OA by suppressing the expression of proinflammatory cytokine genes in synovial MH7A cells by *O*-GlcNAc modification-dependent and -independent mechanisms.

## Introduction

Osteoarthritis (OA) is one of the major joint diseases, which is characterized by cartilage degeneration and osteophyte formation, and accompanied with pain and loss of motion. In the pathogenesis of OA, the aging-related loss of cartilage and the damage to articular cartilage due to accidental injuries and sports are involved [1, 2]. Furthermore, synovitis (synovial inflammation) is associated with the progression of OA [3–5]. During synovial inflammation, synovial cells are activated, and produce proinflammatory cytokines at the local milieu. In this context, it is interesting to note that the severity of OA is correlated with cytokine levels in synovial fluid [6]. Therefore, it is essential to clarify the activation mechanism of synovial cells in the inflammatory synovial tissues for elucidateing the pathogenesis and progression of OA.

Glucosamine (GlcN), a naturally occurring amino monosaccharide, is widely used as a dietary supplement for OA [7–12]. Since GlcN is a component of glycosaminoglycans (GAGs), such as hyaluronic acid (a major constituent of cartilage, connective tissue and synovial fluid), GlcN is estimated to act as a component of GAGs, and contribute to maintaining the structure and function of joints [13, 14]. In addition to its role as a component of GAGs, GlcN exhibits an antiinflammatory action *in vitro* and *in vivo* [15–19]; GlcN suppresses the production of inflammatory mediators such as nitric oxide (NO), prostagrandin (PG) E_2_ and interleukin (IL)-8 by chondrocytes [20–22] and synovial cells [23]. These observations suggest that GlcN exerts the antiinflammatory action on chondrocytes and synovial cells, thereby exhibiting the protective action on OA. However, the detailed mechanism for the antiinflammatory action of GlcN remains poorly understood.

GlcN is incorporated into cells mainly by glucose transporter-2 [24], and converted to uridine diphosphate-*N*-acetylglucosamine (UDP-GlcNAc) via the hexosamine biosynthetic pathway. UDP-GlcNAc is then used for synthesis of glycosaminoglycans. In addition, UDP-GlcNAc is used as a donor substrate by *O*-linked-*N*-acetylglucosamine (*O*-GlcNAc) transferase (OGT), which catalyzes the transfer of *O*-GlcNAc to a hydroxy group of serine and threonine residues in the target proteins [25, 26]. Notably, *O*-GlcNAc modification is one of the principal post-transcriptional modifications, which regulates several cellular functions, including gene expression, signal transductions and subcellular localization of proteins [25–27]. Thus, GlcN is expected to be utilized for the *O*-GlcNAc modification of target proteins, and modulates the cellular functions [28–31].

Previously, we revealed using human umbilical vein endothelial cells (HUVECs) that GlcN suppressed the TNF-α-induced expression of an adhesion molecule (intercellular adhesion molecule-1; ICAM-1) and a chemokine (monocyte chemoattractant protein-1; MCP-1) [32]. Importantly, the suppressive effect of GlcN on ICAM-I and MCP-1 was abrogated by an OGT inhibitor, alloxan [32]. In addition, GlcN inhibited the TNF-α-induced phospholylation of p38 mitogen-activated protein kinase (p38MAPK) and nuclear factor-κB (NF-κB), and the effect of GlcN was also abrogated by alloxan [32]. These observations suggest that GlcN is likely to suppress the expression of ICAM-I and MCP-1, and exert the antiinflammatory action by inhibiting the activation (phospholylation) of p38MAPK and NF-κB via the *O*-GlcNAc modification.

To further elucidate the molecular mechanism for the antiinflammatory actions of GlcN, it is essential to clarify the GlcN-medicated *O*-GlcNAc modification in synovial cells, whose activation is associated with the pathogenesis and progression of OA. Therefore, in the present study, we comprehensively analyzed the effects of GlcN on the gene expression and the *O*-GlcNAc modification in synovial cells by DNA microarray using a human synovial cell line MH7A.

## Materials and Methods

### Reagents

D-Glucosamine hydrochloride was supplied by Koyo Chemical Co., Ltd., (Tokyo, Japan). Human IL-1β was purchased from R & D Systems (Minneapolis, MN, USA); alloxan monohydrate from Sigma Chemical Co. (St. Louis, MO, USA).

### Cells

A human synovial cell line MH7A (RCB1512) [33] was provided by the RIKEN BRC through the National Bio-Resource Project of the MEXT, Japan, and maintained in RPMI-1640 medium (Nacalai Tesque, Kyoto, Japan) containing 10% fetal bovine serum (FBS, Nichirei Biosciences Inc., Tokyo, Japan), 100 units/ml penicillin and 100 μg/ml streptomycin (Nakalai Tesque) at 37°C in 5% CO_2_.

### Quantification of cytokines

MH7A cells (6 × 10^4^ cells/well) were seeded in 24-well plates and cultured overnight at 37°C. After changing the medium to serum- and antibiotics-free RPMI1640, cells were incubated in the presence or absence of GlcN (5mM) for 2.5 h, and then stimulated with 15 pg/ml IL-1β for 13 h. Thereafter, the supernatants were collected, centrifuged at 300 × g for 10 min, and stored at -80°C. Concentrations of IL-6, IL-8 and TNF-α in the supernatants were measured using a DuoSet ELISA Development System (R & D Systems, Minneapolis, MN, USA), according to the manufacturer’s instruction. In some experiments, cells were preincubated with 5 mM alloxan for 30 min, and then incubated with 2 mM alloxan and 5mM GlcN for 2.5 h, followed by stimulation with IL-1β for 13 h. Moreover, to evaluate the dose-dependent effect of GlcN on IL-8 expression, MH7A cells (2 × 10^4^ cells/well) in 48-well plates were preincubated with GlcN (1, 2 or 5 mM) for 2.5 h, and then stimulated with IL-1β.

In a preliminary experiment, MH7A cells were incubated with GlcN (5 mM) for 6–48 h, and then the level of *O*-GlcNAc modification was evaluated by western blotting (as described below). The results indicated that GlcN-mediated *O*-GlcNAc modification reached a maximum at 15 h after the incubation (S1 Fig). Therefore, in this study, MH7A cells were treated with GlcN for total incubation period of 15.5 h.

### Western blot analysis

MH7A cells (3 × 10^5^ cells/well) seeded in 6-well plates were incubated with GlcN and/or alloxan, and stimulated with IL-1β as described above. After washing twice with PBS, cells were harvested and lysed in PBS containing 0.1% Triton X-100, 0.5% sodium dodecyl sulfate (SDS) and protease inhibitor cocktail (Roche Diagnostics, Mannheim, Germany). The lysates were mixed with SDS-polyacrylamide gel electrophoresis (PAGE) sample buffer (62.5 mM Tris-HCl, pH6.8, 2%SDS, 10% glycerol, 0.05% bromphenol blue, and 5% 2-mercaptoethanol), and applied to SDS-PAGE in 8% gels (~15 μg protein/lane). Thereafter, separated proteins were electroblotted onto polyvinylidine fluoride membranes (Immobilon-P; Millipore, Billerica, MA, USA). After incubation with Blocking One (Nacalai Tesque), blots were probed with mouse anti-*O*-GlcNAc monoclonal antibody (CTD110.6; Covance, Berkeley, CA, USA), rabbit anti-ADAMTS (adisintegrin and metalloproteinase with thrombospondin motifs)-1 antibody (ab39194, abcam, Cambredge, UK) or rabbit anti-ADAMTS-12 antibody (H-142, Santa Cruz Biotechnology, Dallas, USA). The membranes were further probed with HRP-conjugated goat anti-mouse IgG/IgM (Chemicon International, Temecula, CA, USA) or HRP-conjugated goat anti-mouse IgG (Chemicon International). The signals were detected using SuperSignal chemiluminescent substrate (Thermo Scientific, Lockford, IL, USA), and quantified using LAS3000 luminescent image analyzer (Fujifilm, Tokyo, Japan) and MultiGauge software (Fujifilm). Thereafter, the blots were stripped using WB Stripping Solution Strong (Naclai Tesque), and glyceraldehyde 3-phosphate dehydrogenase (GAPDH) was detected with mouse anti-GAPDH monoclonal antibody (MAB374, Chemicon Interntional) and HRP-conjugated goat anti-mouse IgG/IgM. The protein contents were determined with BCA Protein Assay Kit (Thermo Scientific).

### Cytotoxicity assay

Cytotoxic effects of GlcN and alloxan were assessed by the release of lactate dehydrogenase (LDH). MH7A cells seeded in 24-well plates were incubated with GlcN and/or alloxan and then stimulated by IL-1β, as described above. Culture supernatants were collected and the LDH activity was measured using an LDH Cytotoxicity Detection Kit (Takara Bio Inc., Shiga, Japan), according to the manufacturer’s instruction. Data was expressed as % of the total LDH activity, which was measured using the supernatants of 1% Triton X-100-treated cell lysates.

### DNA microarray analysis

MH7A cells in 6-well plates were incubated with GlcN and/or alloxan, and stimulated with IL-1β as described above. Thereafter, the cells were harvested, and total RNA was purified using a QIA shredder (QIAGEN, GmbH, Hilden, Germany) and an RNeasy plus mini kit (QIAGEN) according to the manufacturer’s instructions, and stored at -80°C. Single-stranded cDNA was generated from the *in vitro* transcribed cRNA using an Ambion^®^ WT Expression kit (Life Technologies, Carlsbad, CA, USA), and then fragmented and biotinylated with a WT Terminal Labeling Kit (Affymetrix Inc., Santa Clara, CA, USA), according to the manufacturer’s instruction. The labeled cDNA was hybridized with a Human Gene 1.0 ST Array, which contained probe sets of 28,869 annotated genes (Affimetrix^®^ Inc., Santa Clara, CA, USA). The arrays were washed and stained in a GeneChip Fluidics Station 450 (Affymetrix Inc.), according to the manufacturer’s protocol. Thereafter, scanning was carried out with GeneChip Scanner 3000 7G, and scanned images were collected to CEL files by GeneChip operating software (GCOS, Affymetrix Inc.). Then, the background adjustment, normalization and probe summarization of data were processed using robust multiarray average (RMA) algorithm on an Affymetrix Expression Console Software (Affymetrix Inc.). The experiments were repeated 3 times. Data analysis was performed with a GeneSpring GX 12.0 Software (Agilent Technologies, Santa Clara, CA, USA), and statistical significance was determined by Student’s *t*-test. To understand the biological functions of GlcN from the DNA microarray data, ≤1/2-fold downregulated and ≥2-fold upregulated gene by GlcN (*p*<0.05) were selected, and pathway enrichment analysis was performed using a MetaCore Software (Thomson Reuters, New York, NY, USA). The raw microarray data have been deposited in the NCBI Gene Expression Omnibus (GEO; http://ncbi.nlm.nih.gov/geo) and is accessible through GEO Series accession number GSE72575.

### Quantitative real-time reverse transcription (RT)-PCR

MH7A cells (2.5 × 10^6^ cells/well) seeded in 100-mm dishes were cultured overnight, incubated with GlcN and/or alloxan, and stimulated with IL-1β, and then total RNA was isolated from the cells as described above.

First-strand cDNA was synthesized by reverse transcription of total RNA (250 ng) using a ReverTra Ace qPCR RT Master Mix (Toyobo Co., Ltd, Osaka, Japan), according to the manufacturer’s instruction. Quantitative real-time PCR was performed using ABI Prism 7500 Fast Real Time PCR Systems with a Fast SYBR Green Master Mix (Applied Biosystems, Foster City, CA, USA). The sequences and concentrations of primers were shown in S1 Table. Thermal cycling conditions were 20 sec at 95°C, and 40 cycles of 3 sec at 95°C and 30 sec at 60°C. Cycle threshold (Ct) values were calculated by sequence detection system (SDS) software (Applied Biosystems). Results were normalized to the expression of 18S rRNA.

### Statistical analysis

Data were expressed as mean ± standard error. Statistical significance was determined by Student’s *t*-test (GeneSpring GX 12.0 Software) for microarray analysis, and one-way analysis of variance (ANOVA), followed by Turkey’s multiple comparison test (Prism 5, GraphPad Software, San Diego, CA, USA) for other parameters (the data of quantitative RT-PCR and the protein levels). A value of *p*<0.05 was considered to be significant.

## Results

### Effect of GlcN on the IL-8 production and *O*-GlcNAc modification

**Fig 1:**
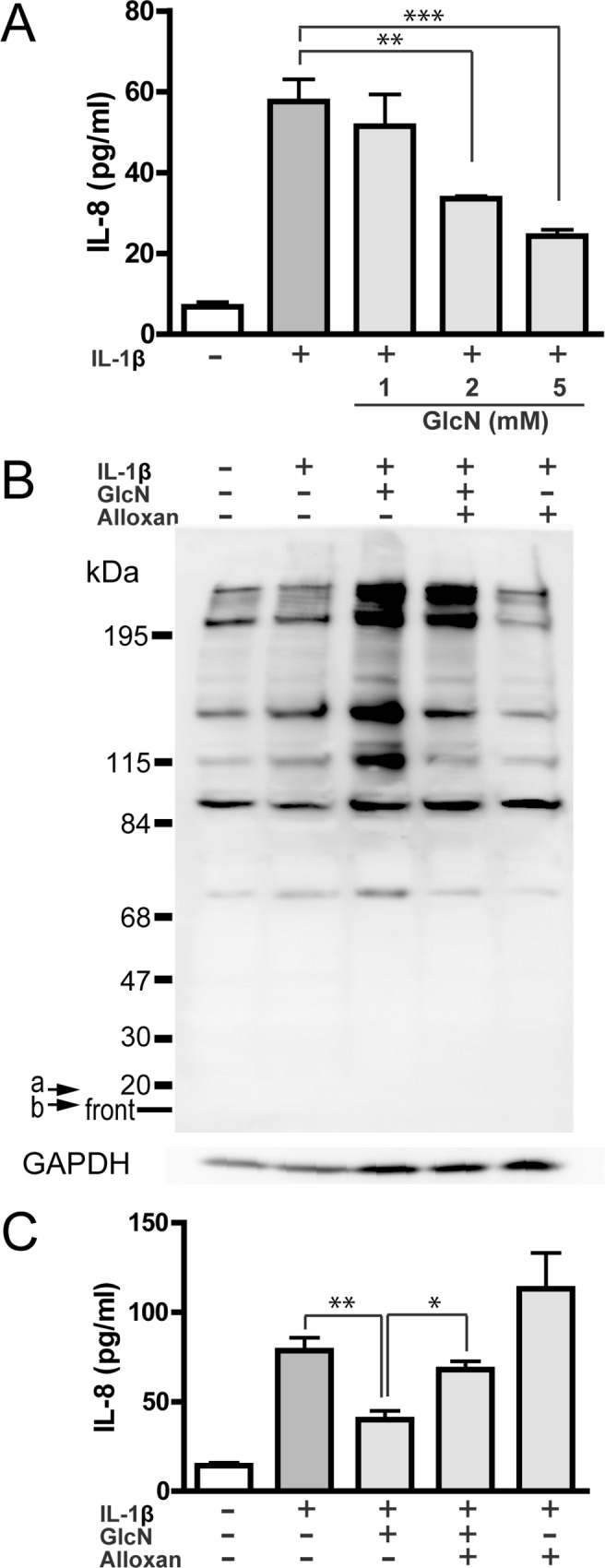
Effect of GlcN on IL-8 production and *O*-GlcNAc modification in MH7A cells. MH7A cells were incubated without (-) or with (+) 15 pg/ml IL-1β for 13h in the absence or presence of GlcN (1, 2 or 5 mM). IL-8 in the culture supernatants was measured by ELISA (A). Data are mean ± S.E. of four to five separate experiments. Values were compared between the absence and presence of GlcN. MH7A cells were incubated without (-) or with (+) 15 pg/ml IL-1β for 13h in the absence or presence of GlcN (5 mM) or GlcN+alloxan. The culture supernatants and cells were recovered, and *O*-GlcNAc-modified proteins and GAPDH (GAPDH) was evaluated by western blotting (B), whereas IL-8 in the culture supernatants was measured by ELISA (C). The image is representative of five separate experiments. Arrows a and b indicated 17 and 8 kDa positions, respectively (B). Data are mean ± S.E. of five separate experiments (C). Values were compared among IL-1β-stimulated cells without or with GlcN or GlcN+alloxan. *p<0.05, **p<0.01, ***p<0.005.

We previously reported that GlcN inhibits the IL-8 production by IL-1β-stimulated synoviocytes isolated from human synovial tissues [23]. To verify the suppressive effect of GlcN on the IL-8 production by human synovial cells, we first examined the dose-dependent effect of GlcN on IL-1β-induced IL-8 production using a human synovial cell line MH7A. IL-1β stimulation markedly increased the IL-8 level. Importantly, GlcN suppressed the IL-8 level in a dose-dependent manner (60% suppression by 5 mM GlcN, Fig 1A).

Next, we investigated the effect of GlcN on *O*-GlcNAc modification in MH7A cells by western blotting using an *O*-GlcNAc specific antibody. Basal level of *O*-GlcNAc modification was detected in resting cells, and IL-1β stimulation did not essentially affect the *O*-GlcNAc modification. Importantly, GlcN enhanced the *O*-GlcNAc modification, and alloxan, an *O*-GlcNAc transferase (OGT) inhibitor, effectively suppressed the GlcN-induced *O*-GlcNAc modification (Fig 1B), although alloxan showed no substantial effect on the *O*-GlcNAc modification in IL-1β-stimulated cells. Notably, alloxan abolished the GlcN-induced suppression of IL-8 production (Fig 1C) as well as the GlcN-induced *O*-GlcNAc modification (Fig 1B). These findings suggest that the suppressive effect of GlcN on the IL-8 production is mediated by the GlcN-induced *O*-GlcNAc modification in MH7A cells. During the stimulation with IL-1β or the incubation with GlcN and alloxan of MH7A cells, LDH release was 5.7~7.6% (4 separate experiments), indicating that all these agents are not essentially cytotoxic.

### Alterations of gene expression by GlcN

We next examined the effect of GlcN on gene expression using microarray. Total RNA was isolated from MH7A cells stimulated with IL-1β in the absence or presence of GlcN, and then subjected to microarray analysis using the Affymetrix Human Gene 1.0 ST Array containing 764,885 probes equivalent to 28,869 well-annotated genes.

First, the effect of IL-1β-stimulation on MH7A cells was examined. IL-1β upregulated 6 genes (≥1.5-fold) and downregulated 1 gene (≤1/1.5-fold) (*p*<0.05) (Table 1). Among the 6 genes, inflammation-related genes, such as TNF receptor superfamily member 9, C3 and IL-6 genes were markedly upregulated by IL-1β (≥2.4-fold). Next, IL-1β-stimulated MH7A cells were incubated with GlcN. Table 2 shows the number of genes, whose expressions were downregulated or upregulated by GlcN. GlcN significantly downregulated 187 genes (≤1/1.5-fold) and 40 genes (≤1/2-fold), and upregulated 194 genes (≥1.5-fold) and 53 genes (≥2-fold) (*p*<0.05). The genes downregulated and upregulated by GlcN are listed in S2 Table and S3 Table, respectively, as gene symbols. In addition, their detailed data from the microarray analysis are described in S4 Table.

**Table 1:** Genes regulated by IL-1β.

Gene symbols	Regulation (Up or Down)	Fold change	Gene tytles
TNFRSF9	Up	3.5	tumor necrosis factor receptor superfamily, member 9
C3	Up	2.8	complement component 3
IL6	Up	2.4	interleulin-6
NFKBIA	Up	1.8	I-kappa-B-alpha
CLDN1	Up	1.6	claudin-1
NFE2L3	Up	1.6	nuclear factor 3
RNU4-2	Down	0.51	RNA U4 small nuclear 2

DNA microarray experiments were repeated three times. Gene expression levels were compared between IL-1β-unstimulated and stimulated cells. Fold change was calculated as the ratio of IL-1β-stimulated signal to unstimulated signal. Significantly upregulated or downregulated genes (≥1.5-fold or ≤1/1.5-fold, *p*<0.05) are listed.

**Table 2:** Number of GlcN-regulated genes.

**Downregulated (GlcN/Control)**	**Number of genes**
≤1/1.5-fold	187
≤1/2-fold	40
**Upregulated (GlcN/Control)**	**Number of genes**
≥1.5-fold	194
≥2-fold	53

Gene expression levels were compared between the IL-1β-stimulated cells without and with GlcN treatment. Fold change was calculated as the ratio of GlcN-treated signal (GlcN) to nontreated signal (Control). Number of genes significantly downregulated (≤1/1.5-fold or ≤1/2-fold, *p*<0.05) or upregulated (≥1.5-fold or ≥2-fold, *p*<0.05) by GlcN are listed.

Next, GlcN-regulated 93 genes (40 downregulated genes, ≤1/2-fold and 53 upregulated genes, ≥2-fold; Table 2) were subjected to pathway analysis as shown in Table 3. The results indicated that GlcN-regulated genes are mainly categorized into 4 immune response-related pathways and 4 amino acid metabolism in MH7A cells.

**Table 3:** Pathway analysis of the GlcN-regulated genes.

Pathways	*p*-value
Immune response IL-1 signaling pathway	0.00033
Glycine, serine, cysteine and threonine metabolism	0.00053
Glycine, serine, cysteine and threonine metabolism/Rovent version	0.00056
Apoptosis and survival endoplasmic reticulum stress response pathway	0.00057
Immune response IL-6 signaling pathway	0.0036
Immune response signaling pathway mediated by IL-6 and IL-1	0.0039
Methionine metabolism	0.005
Immune response MIF in innate immunity response	0.0068
Development growth hormone signaling via PI3K/AKT and MAAPK cascade	0.0075
Methionine-cysteine-glutamate metabolism	0.0078

GlcN-downregulated 40 (≤1/2-fold, *p*<0.05) and upregulated 53 (≥2-fold, *p*<0.05) genes in S1 and S2 Tables were subjected to enrichment pathway analysis. The top 10 pathways are shown in the order of *p*-values.

Furthermore, the effect of GlcN on the expression of proinflammatory and antiinflammatory cytokine genes were evaluated, based on the microarray data. Table 4 shows that the expression of proinflammatory cytokine genes (such as IL-6, IL-24 and TNF-α genes) was significantly downregulated by GlcN. The expression of IL-8 mRNA was slightly downregulated by GlcN (Table 4) (*p* = 0.18), although the IL-1β-induced increase of IL-8 was significantly inhibited by GlcN (Fig 1A). In contrast, the expression of antiinflammatory cytokine genes (such as IL-4, IL-10, IL-13 and TGF-β) was not substantially affected by GlcN. Thus, GlcN is likely to exert an antiinflammatroy action on synovial cells by suppressing the proinflammatory genes rather than the antiinflammatory genes.

**Table 4:** Effect of GlcN on the expression of cytokine genes.

**Proinflammatory cytokines**		
**Genes**	**Fold change (GlcN/Control)**	***p*-value**
IL-1β	0.9	0.23
IL-6	0.46	0.02*
IL-8	0.76	0.18
IL-12	1.03	0.61
IL-15	1.09	0.57
IL-17A	1.02	0.86
IL-17B	1	0.99
IL-17C	1.06	0.59
IL-17D	1.09	0.44
IL-17F	1.01	0.68
IL-18	0.84	0.22
IL-21	1.07	0.22
IL-22	1.08	0.16
IL-24	0.54	0.02*
TNF-α	0.8	0.04*
IFNγ	1.03	0.32
G-CSF	0.96	0.65
**Antiinflammatory cytokines**		
**Genes**	**Fold change (GlcN/Control)**	***p*-value**
L-4	0.99	0.73
IL-10	1.05	0.27
IL-13	1.03	0.72
TGF-β	10.84	0.08

Pronflammatory and antiinflammatory cytokines were selected, and the expression was compared between the IL-1β-stimulated cells without and with GlcN treatment. Fold change was calculated as the ratio of GlcN-treated signal (GlcN) to nontreated signal (Control).

*p<0.05.

### Effect of alloxan on GlcN-induced alterations of gene expression

**Fig 2:**
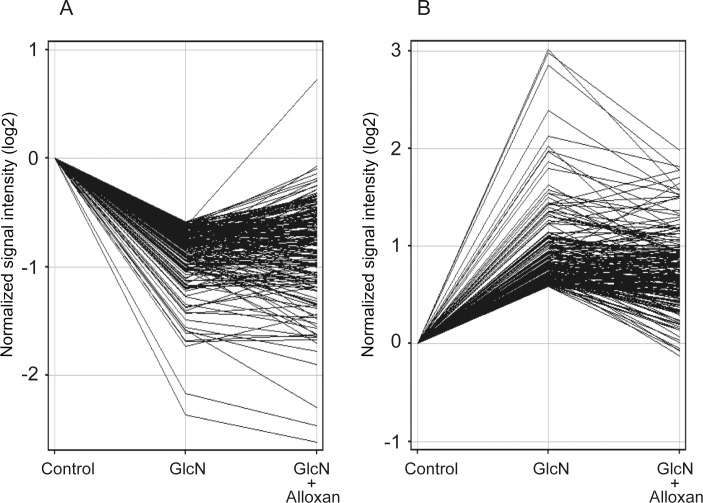
Effect of alloxan on GlcN-mediated change of gene expression. The effect of alloxan on the expression of 187 downregulated genes (≤1/1.5-fold, A) or 194 upregulated genes (≥1.5-fold, B) by GlcN (S2 and S3 Tables) was analyzed by GeneSpring Software using IL-1β-stimulated cells without (Control) or with GlcN or GlcN+alloxan. Data are average of three separate experiments, and gene expression was expressed as a ratio relative to Control.

GlcN is incorporated into the cells and utilized for the *O*-GlcNAc modification of target proteins by the action of OGT [25, 28, 30–32, 34]. Thus, to understand a role of *O*-GlcNAc modification in GlcN-induced alterations of gene expression, the effect of OGT inhibitor, alloxan, was evaluated. Fig 2 shows the effects of alloxan on the expression of GlcN-downregulated (≤1/1.5-fold) and upregulated (≥1.5-fold) genes (shown in Table 2). Among the 187 downregulated genes, the expression of 100 genes (53.4%) was restored by alloxan, based on the ratio (<1.0) (the changes of mRNA expression in the presence of both GlcN and alloxan/the changes in the presence of GlcN) (Fig 2A, S5 Table). Moreover, among 194 upregulated genes, the expression of 139 genes (71.6%) was restored by alloxan, based on the ratio (<1.0) (Fig 2B, S6 Table). These observations likely suggest that among the GlcN-downregulated or upregulated genes, the expression of more than 50% of the genes is mediated by *O*-GlcNAc modification.

**Fig 3:**
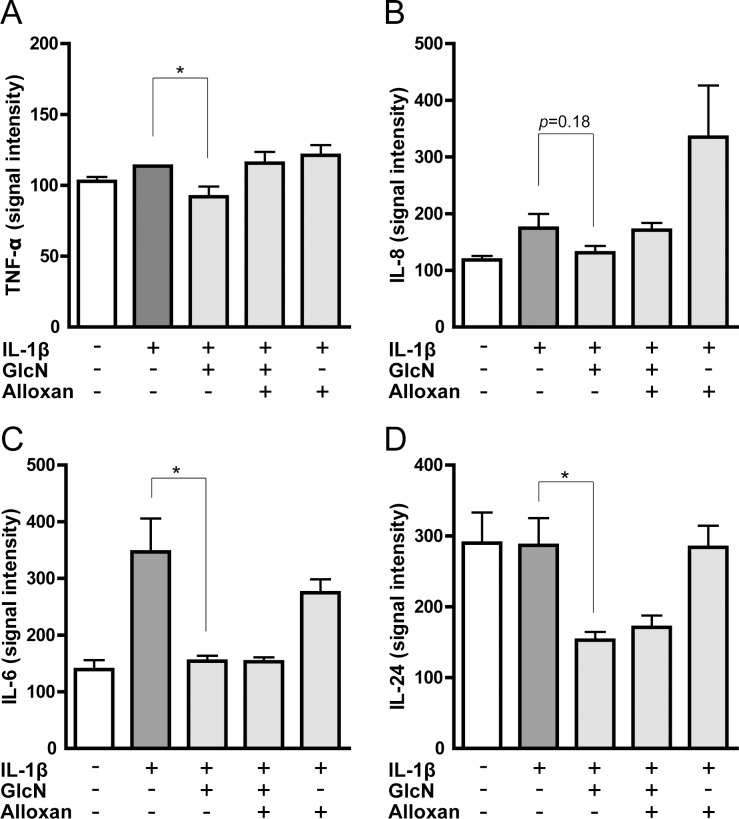
Effects of GlcN and alloxan on the expression of TNF-α, IL-6, IL-8 and IL-24 genes analyzed by microarray. Expression of TNF-α (A), IL-8 (B), IL-6 (C) and IL-24 (D) genes was illustrated based on the microarray data using MH7A cells incubated without (-) or with (+) IL-1β in the absence or presence of GlcN or GlcN+alloxan. Data are mean ± S.E. of three separate experiments. Values were compared between IL-1β-stimulated cells without and with GlcN. *p<0.05.

**Fig 4:**
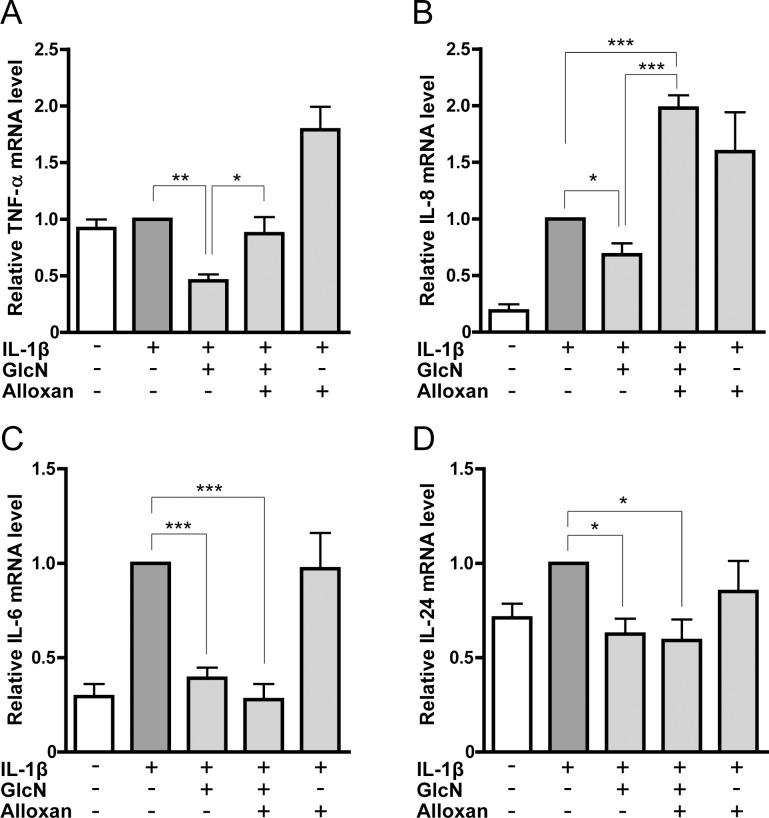
Effects of GlcN and alloxan on the expression of TNF-α, IL-6, IL-8 and IL-24 genes analyzed by quantitative RT-PCR. Expression of TNF-α (A), IL-8 (B), IL-6 (C) and IL-24 (D) genes was illustrated based on the quantitative RT-PCR analysis using MH7A cells incubated without (-) or with (+) IL-1β in the absence or presence of GlcN or GlcN+alloxan. Data are mean ± S.E. of four to eight separate experiments. Values were compared among IL-1β-stimulated cells without or with GlcN, or GlcN+alloxan. *p<0.05, **p<0.01, ***p<0.005.

We further focused on the effect of alloxan on the mRNA expression of proinflammatory cytokines. The microarray data indicated that the suppressive effect of GlcN on TNF-α and IL-8 genes was abrogated by alloxan (Fig 3A and 3B), whereas that of IL-6 and IL-24 genes was not affected by alloxan (Fig 3C and 3D). Similarly, quantitative RT-PCR indicated that alloxan abrogated the GlcN-downregulated expression of TNF-α and IL-8 mRNA but not IL-6 and IL-24 mRNA (Fig 4). Low threshold cycle (Ct) values of each gene were shown in supplemental table (S7 Table).

**Fig 5:**
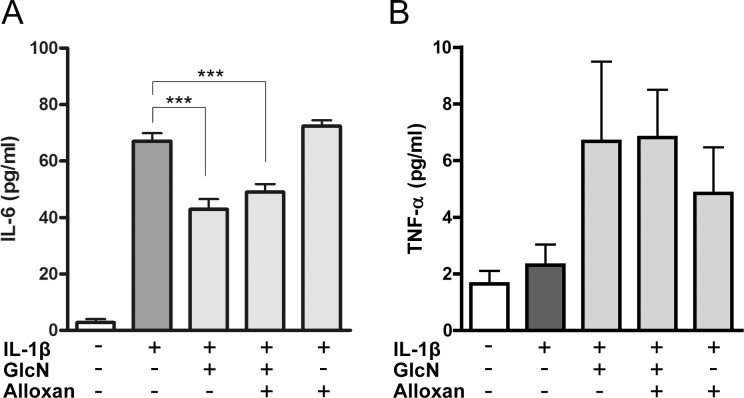
Effects of GlcN and alloxan on the levels of IL-6 and TNF-α. MH7A cells were incubated without (-) or with (+) 15 pg/ml IL-1β for 13h in the absence or presence of GlcN (5 mM) or GlcN+alloxan. IL-6 (A) and TNF-α (B) in the culture supernatants was measured by ELISA. Data are mean ± S.E. of seven to eight separate experiments. Values were compared among IL-1β-stimulated cells with or without GlcN, or GlcN+alloxan. ***p<0.005.

Finally, we examined the effects of GlcN and alloxan on the levels of IL-6 and TNF-α produced by IL-1β-stimulated MH7A cells. Consistent with the data of microarray and quantitative RT-PCR (Figs 3C and 4C), GlcN suppressed the IL-6 level; however, alloxan did not restore the suppressed level of IL-6 (Fig 5A). In contrast, the level of TNF-α was not essentially affected by GlcN and alloxan (Fig 5B), although GlcN suppressed the level of TNF-α mRNA, and alloxan restored the suppressed level of TNF-α mRNA (Fig 4). Based on the effects of GlcN and alloxan on IL-8 and IL-6 (Figs 1, 3, 4 and 5), GlcN likely suppresses the mRNA and protein levels of proinflammatory cytokines via *O*-GlcNAc modification-dependent (alloxan-restorable) and -independent (alloxan-unrestorable) mechanisms.

## Discussion

In this study, to elucidate the molecular mechanism involved in the GlcN-medicated regulation of synovial cell activation, we comprehensively analyzed the effect of GlcN on the gene expression in a human synovial cell line MH7A by DNA microarray. In the previous studies, synovial cells (including MH7A cells) [35, 36] and chondrocytes [37] were stimulated with the high concentrations of IL-1β (1–20 ng/ml). However, the progression of OA is associated with the chronic and low-grade synovial inflammation [38]. Thus, we stimulated MH7A cells with a low concentration of IL-1β (15 pg/ml). Under our experimental condition with 15 pg/ml IL-1β, the expression of only 7 genes was affected in MH7A cells (Table 1), whereas the expression of 2813 genes are affected by the high concentration of 10 ng/ml IL-1β in chodrocytes [37]. Moreover, in the present study, we confirmed that the gene expression of proinflammatory cytokines (such as IL-6 and IL-8) is significantly upregulated in MH7A cells by IL-1β (Fig 4). Thus, we believe that a MH7A cell line can be used as a model for evaluating synovial inflammation. Notably, 5 mM GlcN significantly downregulates the expression of 187 genes (≤1/1.5-fold) and upregulates the expression of 194 genes (≥1.5-fold) in IL-1β-stimulated MH7A cells. Interestingly, pathway analysis indicated that among the 10 pathways, into which the GlcN-regulated genes are categorized, the 4 pathways are immune-related.

To date, mechanisms for the action of GlcN on OA have been mainly elucidated using chondrocytes, because OA is characterized by the destruction of articular cartilage. DNA microarray analysis using rat chondrocytes revealed that GlcN inhibits the IL-1β-induced gene expression of cytokines, chemokines, growth factors, and PGE_2_- and NO-synthesizing enzymes [37]. In addition, DNA microarray analysis using a human chondrocyte cell line (lbpva55) indicated that GlcN downregulates the expression of several genes, including TNF-α receptor genes [39]. Importantly, in the present study, GlcN suppressed the mRNA and protein levels of proinflammatory cytokines (such as IL-6 and IL-8) in a human synovial cell line MH7A. Together, these observations indicate that GlcN possibly exerts a protective action on the joints of OA with cartilage destruction and synovial inflammation, by suppressing the expression of inflammatory molecules (such as cytokines, chemokines, enzymes and receptors) in not only chondrocytes but also synovial cells.

To further investigate the molecular mechanism for the action of GlcN on the gene expression, we utilized alloxan, an OGT inhibitor, which inhibits the *O*-GlcNAc modification. The results indicated that among the GlcN-downregulated or -upregulated genes, the expression of more than 50% of the genes is mediated by *O*-GlcNAc modification, based on the effects of alloxan. Moreover, GlcN suppressed the expression of proinflammatory cytokine genes but not antiinflammatory genes (Table 4). Thus, we focused on the effect of alloxan on the GlcN-induced suppression of proinflammatory cytokines (IL-6, IL-8, IL-24 and TNF-α, and revealed that alloxan abrogated the GlcN-induced downregulation of TNF-α and IL-8 mRNA but not IL-6 and IL-24 mRNA (Figs 3 and 4). Furthermore, we confirmed that GlcN suppressed the protein levels of IL-8 and IL-6, and alloxan restored the suppressed levels of IL-8 but not IL-6. Together these observations suggest that GlcN suppresses the expression of proinflammatory cytokines via *O*-GlcNAc modification-dependent (alloxan-restorable) and -independent (alloxan-unrestorable) mechanisms. Of interest, the level of TNF-α was too low, and the effects of GlcN and alloxan could not be detected in our experiments (Fig 5B), although GlcN suppressed the level of TNF-α mRNA, and alloxan restored the suppressed level of TNF-α mRNA (Fig 4A). As shown in Fig 1B, the bands corresponding to TNF-α (17kDa) and IL-8 (8 kDa) could not be detected. Thus, in our experiment, GlcN is unlikely to induce the *O*-GlcNAc modification of TNF-α and IL-8. Consistent with our results, it has been reported that IL-1β significantly upregulates the level of TNF-α mRNA in MH7A cells, but TNF-α protein can not be measured in the supernatants [36], suggesting a discrepancy between mRNA and protein expression in MH7A cells.

Transcription of IL-6, IL-8, IL-24 and TNF-α genes is controlled by multiple transcription factors, such as NF-κB [40–43]. Interestingly, the functions of NF-κB are regulated by *O*-GlcNAc modification [44, 45]. In this context, GlcN inhibits the TNF-α-induced chemokine expression by rat smooth muscle cells via the *O*-GlcNAc modification of NF-κB p65 [46] Moreover, we previously revealed that GlcN inhibits the expression of chemokine and adhesion molecule by endothelial cells via *O*-GlcNAc modification of NF-κB p65 [32]. In this study, we confirmed that GlcN enhances the *O*-GlcNAc modification of NF-κB p65 (S2 Fig) but suppresses the expression of cytokines in MH7A cells. These observations likely suggest that the expression of proinflammatory cytokine genes may be regulated by the mechanism involving the *O*-GlcNAc-modification of NF-κB.

In addition to the suppression of proinflammatory cytokines, the present results demonstrated that GlcN downregulated the expression of genes for ADAMTSs (adisintegrin and metalloproteinase with thrombospondin motifs) (S2 Table). ADAMTSs degrade the components of cartilage, such as aggrecan and COMP (cartilage oligomeric matrix protein), and contribute to the structural damage in OA [47–50]. In the present study, quantitative RT-PCR and western blot analyses indicated that GlcN substantially suppresses the mRNA and protein levels of ADAMTS-1 and -12 (S3 Fig); however, alloxan could not restore the GlcN-induced suppression of ADAMTS-1 and -12 gene expression (S5 Table). These observations suggest that GlcN may exhibit the protective action on cartilage in OA by downregulating the ADAMTS genes (cartilage matrix degrading enzymes) in synoviocytes, possibly via an *O*-GlcNAc modification-independent mechanism.

In conclusion, to elucidate the molecular mechanisms for the action of GlcN on synovial cells, we comprehensively analyzed the effect of GlcN on gene expression using a human synovial cell line MH7A by DNA microarray. The results indicated that GlcN suppresses the expression of proinflammatory cytokine genes (such as IL-6, IL-24 and TNF-α genes). Moreover, we confirmed that GlcN-mediated *O*-GlcNAc modification is involved in the GlcN-induced suppression of mRNA and protein levels of IL-8 but not IL-6. Thus, GlcN likely exerts an antiinflammatroy action on synovial cells in OA by suppressing the expression of proinflammatory cytokine genes via *O*-GlcNAc modification-dependent and -independent mechanisms, and exhibits the protective action on OA. To further clarify the action of GlcN on the gene expression, the molecules governing *O*-GlcNAc modification-dependent or -independent mechanism should be identified in the future.

## Supporting Information

S1 Fig Time-dependent effect of GlcN on *O*-GlcNAc modification. MH7A cells were incubated with GlcN (5 mM) for 6, 15, 24 or 48 h. Cells were recovered and *O*-GlcNAc-modified proteins were evaluated by western blotting. All detectd bands were quantified, and relative *O*-GlcNAc modification levels were expressed.
(TIF)

S2 Fig Effect of GlcN on *O*-GlcNAc modification of NF-κB p65. MH7A cells were incubated without (-) or with (+) 15 pg/ml IL-1β for 13h in the absence (-) or presence (+) of GlcN (5 mM). Cell lysates (100 μg) were incubated with mouse anti-p65 antibody (200 ng, F-6; Santa Cruz Biotechnology Inc.) for 4h, and then further incubated with protein A/G PLUS-agarose (5μl; Santa Cruz Biotechnology Inc.) for overnight. After washing with three times by TBS containing 0.02% NP-40, binding proteins on the agarose beads were eluted with the SDS-PAGE sample buffer solution for at 65°C for 15 min. Samples were applied to SDS-PAGE on 10% gels, and blotted on PVDF membranes. After blocking, blots were probed with mouse anti-*O*-GlcNAc antibody and HRP-conjugated goat anti-mouse IgG. The signals were detected and quantified. After stripping the antibodies, further detected p65 using rabbit anti-p65 antibody (D14E12; Cell Signaling Technology, Danvers, MA, USA). Data are mean ± S.E. of three separate experiments. *p<0.05.
(TIF)

S3 Fig Effect of GlcN on mRNA and protein expression of ADAMTSs. MH7A cells were incubated without (-) or with (+) 15 pg/ml IL-1β for 13h in the absence (-) or presence (+) of GlcN (5 mM). The expression of ADAMTS-1, -6 and -12 mRNA was quantified by real-time RT-PCR (A). mRNA expression was expressed as a relative to IL-1β stimulation alone. Data are mean ± S.E. of five separate experiments. Cells were recovered, and ADAMTS-1 (B) and ADAMTS-12 (C) and GAPDH (GAPDH) were evaluated by western blotting. Blots shown are representative of eight separate experiments. Blots were quantified using an image analyzer. Protein expression was expressed as a relative to IL-1β stimulation alone. Data are mean ± S.E. of eight separate experiments. *p<0.05, **p<0.01.
(TIF)

S1 Table Primers used for real-time PCR. (PDF)

S2 Table Genes downregulated by GlcN. (PDF)

S3 Table Genes upregulated by GlcN. (PDF)

S4 Table Detailed data from DNA microarray analysis. (PDF)

S5 Table GlcN-downregulated genes, whose expression was modulated by alloxan. (PDF)

S6 Table GlcN-upregulated genes, whose expression was modulated by alloxan. (PDF)

S7 Table Ct values from quantitative real-time RT-PCR. (PDF)
