# Supplementary figures and images for: Glucosamine Downregulates the IL-1β-Induced Expression of Proinflammatory Cytokine Genes in Human Synovial MH7A Cells by O-GlcNAc Modification-Dependent and -Independent Mechanisms
Source: PLoS One. 2016 Oct 24;11(10):e0165158. doi: 10.1371/journal.pone.0165158 (PMC5077170; doi:10.1371/journal.pone.0165158)

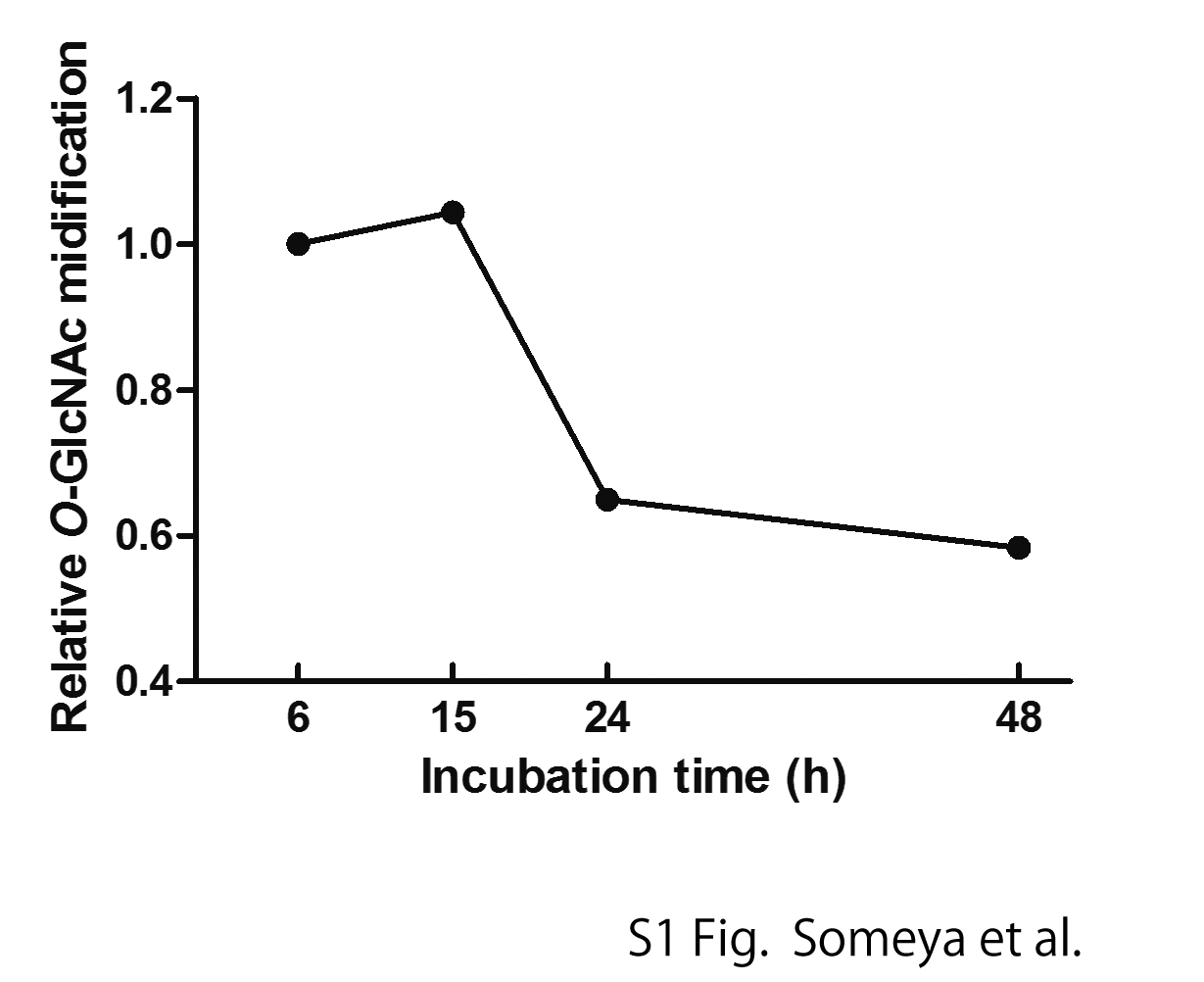

Supplement: S1 Fig — MH7A cells were incubated with GlcN (5 mM) for 6, 15, 24 or 48 h. Cells were recovered and O-GlcNAc-modified proteins were evaluated by western blotting. All detectd bands were quantified, and relative O-GlcNAc modification levels were expressed. (TIF) [file pone.0165158.s001.tif]

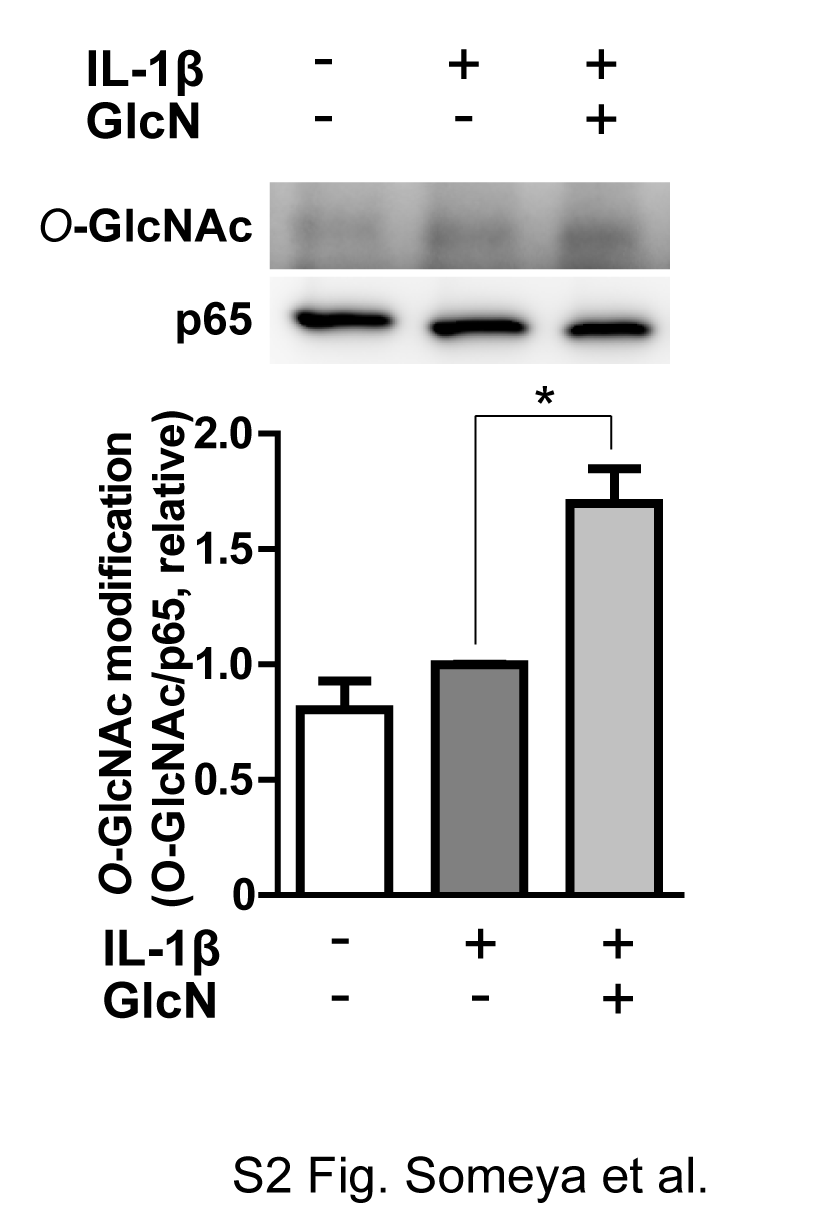

Supplement: S2 Fig — MH7A cells were incubated without (-) or with (+) 15 pg/ml IL-1β for 13h in the absence (-) or presence (+) of GlcN (5 mM). Cell lysates (100 μg) were incubated with mouse anti-p65 antibody (200 ng, F-6; Santa Cruz Biotechnology Inc.) for 4h, and then further incubated with protein A/G PLUS-agarose (5μl; Santa Cruz Biotechnology Inc.) for overnight. After washing with three times by TBS containing 0.02% NP-40, binding proteins on the agarose beads were eluted with the SDS-PAGE sample buffer solution for at 65°C for 15 min. Samples were applied to SDS-PAGE on 10% gels, and blotted on PVDF membranes. After blocking, blots were probed with mouse anti-O-GlcNAc antibody and HRP-conjugated goat anti-mouse IgG. The signals were detected and quantified. After stripping the antibodies, further detected p65 using rabbit anti-p65 antibody (D14E12; Cell Signaling Technology, Danvers, MA, USA). Data are mean ± S.E. of three separate experiments. *p<0.05. (TIF) [file pone.0165158.s002.tif]

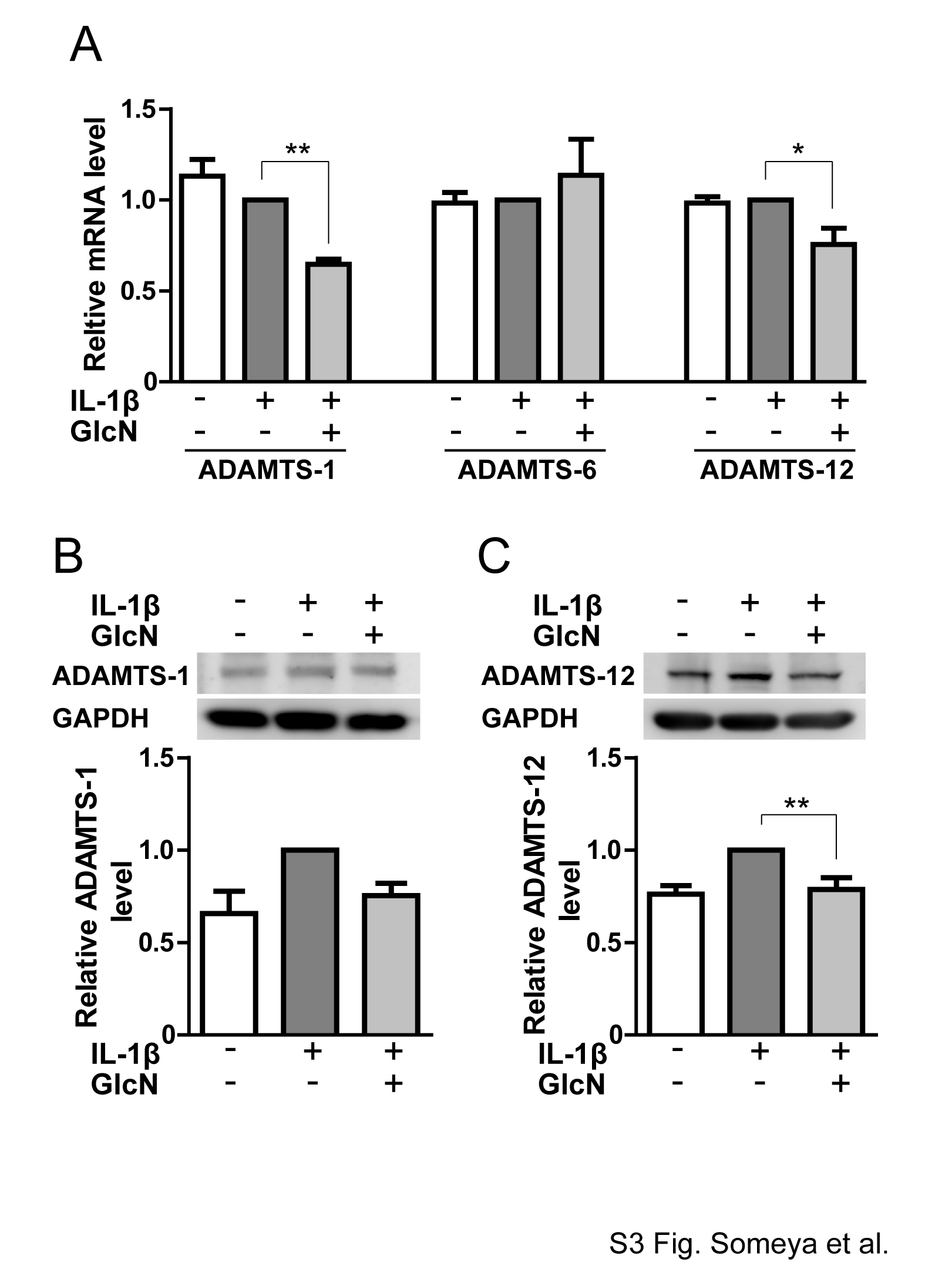

Supplement: S3 Fig — MH7A cells were incubated without (-) or with (+) 15 pg/ml IL-1β for 13h in the absence (-) or presence (+) of GlcN (5 mM). The expression of ADAMTS-1, -6 and -12 mRNA was quantified by real-time RT-PCR (A). mRNA expression was expressed as a relative to IL-1β stimulation alone. Data are mean ± S.E. of five separate experiments. Cells were recovered, and ADAMTS-1 (B) and ADAMTS-12 (C) and GAPDH (GAPDH) were evaluated by western blotting. Blots shown are representative of eight separate experiments. Blots were quantified using an image analyzer. Protein expression was expressed as a relative to IL-1β stimulation alone. Data are mean ± S.E. of eight separate experiments. *p<0.05, **p<0.01. (TIF) [file pone.0165158.s003.tif]
