# Supplementary material for: Glucosamine Downregulates the IL-1β-Induced Expression of Proinflammatory Cytokine Genes in Human Synovial MH7A Cells by O-GlcNAc Modification-Dependent and -Independent Mechanisms
Source: PLoS One. 2016 Oct 24;11(10):e0165158. doi: 10.1371/journal.pone.0165158 (PMC5077170; doi:10.1371/journal.pone.0165158)
Supplement: S1 Table — (PDF) [file pone.0165158.s004.pdf]

S1 Table. Primers used for real-time PCR

Genes	Forward primers	Reverse primers	Concentrations (nM)
IL-6	5’-GATGGCTGAAAAAGATGGATG -3’	5’-CAGCTCTGGCTTGTTCCTCAC-3’	200
IL-8	5’-ACACTGCGCCAACACAGAAAT-3’	5’-ACACTGCGCCAACACAGAAAT-3’	100
IL-24	5’-AGAATTGAGGCTGCTTGGGA-3’	5’-AAGGGTCTGCTGGCTAAAGTC-3’	200
TNF-α	5’-CTCTCTAATCAGCCCTCTGGC-3’	5’-ATGAGGTACAGGCCCTCTGAT-3’	250
ADANTS-1	5’-GAACAAAACCGACAGAAAGCA -3	5’- TGTCACATTCCCTCATCGTG -3’	200
ADAMTS-6	5’-GTCAGAATGCTCAGCTACTTGTGC-3	5’-TTGTTCTCCATCTTTCCCTCTG-3’	200
ADAMTS-12	5’-ACAGGCGAGCGCTTTATTTC -3	5’-AAAGGTTTGCAAGCCAGCTC-3’	200
18S	5’-CGGCTACCACATCCAAGGAA-3’	5’-GCTGGAATTACCGCGGCT-3’	150
