# Supplementary material for: Glucosamine Downregulates the IL-1β-Induced Expression of Proinflammatory Cytokine Genes in Human Synovial MH7A Cells by O-GlcNAc Modification-Dependent and -Independent Mechanisms
Source: PLoS One. 2016 Oct 24;11(10):e0165158. doi: 10.1371/journal.pone.0165158 (PMC5077170; doi:10.1371/journal.pone.0165158)
Supplement: S3 Table — (PDF) [file pone.0165158.s006.pdf]

S3 Table. Genes upregulated by GlcN

Gene symbols (Fold change)				
INHBE (8.14)	C12orf39 (7.94)	BEST1 (7.26)	SLC6A9 (5.27)	DDIT3 (4.38)
IL20RB (4.08)	WARS (3.95)	ALDH1L2 (3.91)	SLC7A11 (3.64)	ASNS (3.48)
SLFN5 (3.09)	NUPR1 (3.00)	CHAC1 (2.99)	TRIB3 (2.93)	HERPUD1 (2.93)
GPR1 (2.84)	PCK2 (2.74)	SDR42E1 (2.71)	ERO1LB (2.70)	ULBP1 (2.70)
CEBPB (2.64)	PHGDH (2.57)	PSAT1 (2.57)	ZNF619 (2.56)	FAM129A (2.52)
NSAP11 (2.48)	AARS (2.48)	CCPG1 (2.47)	DNAJC3 (2.40)	SLC10A5 (2.36)
SESN2 (2.35)	GABRG1 (2.34)	DDR2 (2.22)	WIPI1 (2.21)	SLC3A2 (2.20)
C17orf75 (2.16)	CTH (2.15)	SDF2L1 (2.15)	HYOU1 (2.14)	SLC1A4 (2.14)
MOCOS (2,14)	DDIT4 (2.13)	CEBPG (2.11)	PDIA4 (2.11)	TUBGCP3 (2.07)
TSEN15 (2.06)	HDDC3 (2.06)	SLC35B1 (2.06)	CBS (2.05)	SLC16A14 (2.05)
MTHFD2 (2.05)	HCG8 (2.03)	GOLPH3L (2.01)	GDF15 (2.00)	PDE5A (2.00)
KLHL5 (1.99)	TMEM50B (1.99)	SLC16A12 (1.97)	C5orf28 (1.97)	GTPBP2 (1.94)
CDK2AP2 (1.92)	TCP11L2 (1.92)	GARS (1.92)	KRCC1 (1.92)	IFT46 (1.92)
THG1L (1.90)	SLC33A1 (1.90)	PXK (1.90)	ZNF204P (1.89)	SLC7A5 (1.89)
DCP1B (1.87)	DNAJB9 (1.86)	SEL1L (1.86)	TUBE1 (1.85)	SGK3 (1.85)
C11orf83 (1.84)	HSPA5 (1.83)	PGBD1 (1.83)	CEP120 (1.82)	SLC35A1 (1.82)
XPOT (1.81)	ZNF187 (1.81)	FCRLA (1.80)	C9orf150 (1.80)	ZC3H6 (1.80)
CARS (1.78)	MANF (1.77)	IL1RL1 (1.77)	ZNF22 (1.77)	SEC11C (1.77)
RNF125 (1.76)	ZNF70 (1.76)	SLC30A4 (1.75)	RHOB (1.74)	DUSP12 (1.74)
CRELD1 (1.74)	SARS (1.74)	DPY19L4 (1.73)	UHRF1BP1 (1.73)	YARS (1.73)
LMO4 (1.73)	LARP6 (1.72)	ICT1 (1.72)	TTC17 (1.72)	GPT2 (1.72)
DCLRE1A (1.72)	NUCB2 (1.71)	VEGFA (1.71)	MARS (1.70)	UBE2D4 (1.70)
CYLD (1.70)	C10orf57 (1.70)	IBTK (1.70)	LPHN3 (1.69)	COQ5 (1.69)
IFRD1 (1.69)	C6orf48 (1.69)	TRPM6 (1.67)	LOC100129534 (1.67)	CSGALNACT2 (1.67)
ALOX5AP (1.66)	TSC22D3 (1.66)	DUSP16 (1.65)	PRPSAP1 (1.65)	LOC728819 (1.64)
OR2A4 (1.64)	MDGA2 (1.63)	HEATR5A (1.63)	ARHGEF2 (1.63)	NEK3 (1.63)
MAP1B (1.63)	AAAS (1.63)	ASS1 (1.63)	NEK4 (1.62)	AP1S3 (1.62)
C9orf91 (1.62)	XBP1 (1.62)	NCRNA00171 (1.61)	ZNF311 (1.61)	FBXO8 (1.61)
TRAF6 (1.61)	JMY (1.60)	TRIM16L (1.60)	MKNK2 (1.60)	ZFP62 (1.59)
JDP2 (1.59)	ALPK1 (1.59)	DNAJC16 (1.59)	RAB39B (1.59)	PRKAB2 (1.59)
OR2A9P (1.58)	DPH5 (1.58)	TES (1.58)	CLGN (1.58)	ANKRD46 (1.58)
PPP1R15A (1.58)	PAN2 (1.57)	HIST2H2AB (1.57)	SHMT2 (1.57)	ULK2 (1.57)
POLR3B (1.56)	FKBP9L (1.56)	ATPAF1 (1.55)	PRKCQ (1.55)	SNAPIN (1.55)
WDR25 (1.55)	ZNF445 (1.55)	CSF2RA (1.54)	KLHL24 (1.54)	TMEM136 (1.54)
ZKSCAN3 (1.54)	LOC554249 (1.54)	ANKRD5 (1.54)	GOT1 (1.54)	HSPC157 (1.53)
SRD5A3 (1.53)	EDEM1 (1.52)	PPAPDC1B (1.52)	HEY1 (1.52)	ZNF581 (1.52)
TRAM1 (1.52)	B4GALT7 (1.52)	ACTA2 (1.51)	KIAA1529 (1.51)	RHBDD1 (1.51)
RAD1 (1.51)	NCOA7 (1.51)	ATPBD4 (1.51)	BCAT1 (1.50)	TMEM231 (1.50)
SCN9A (1.50)	CCDC101 (1.50)	C17orf60 (1.50)	DNAJB11 (1.50)	

GlcN-upregulated 194 genes (≥1.5-fold, $p<0.05$) are listed. Fold change was calculated as the ratio of GlcN-treated signal to nontreated signal.
