# Supplementary material for: Glucosamine Downregulates the IL-1β-Induced Expression of Proinflammatory Cytokine Genes in Human Synovial MH7A Cells by O-GlcNAc Modification-Dependent and -Independent Mechanisms
Source: PLoS One. 2016 Oct 24;11(10):e0165158. doi: 10.1371/journal.pone.0165158 (PMC5077170; doi:10.1371/journal.pone.0165158)
Supplement: S4 Table — (PDF) [file pone.0165158.s007.pdf]

S4 Table. Detailed data from the DNA microarray analysis

Notes : Created from Advanced Analysis operation: significance Analysis.

#Entitylist : SOMEYA GlcN n=3 Filtered on Expression 24216

#Interpretation : MH7A GlcN

#Experiment: SOMEYA n=3

#corrected p-value cut-off:0.05

#Fold change cut-off:1.5

#Selected Test : T Test unpaired

#p-value computation: Asymptotic

#Multiple Testing Correction: No Correction

#

Technology : Affymetrix.ExonExprChip.HuGene-1_0-st-v1_na31_hg19_2010-09-03

Owner : gxuser

Created On : Fri Nov 04 10:54:55 JST 2011

Transcripts Cluster Id	p-value (Control vs GlcN)	Gene Symbol	Gene Title	fold change	regulation	[Resting](raw)	[Control](raw)	[GlcN](raw)	[GlcN + Allo](raw)	[Alloxan](raw)	Effect of Alloxan	[Resting](normalized)	[Control](normalized)	[GlcN](normalized)	[GlcN + Allo](normalized)	[Alloxan](normalized)
8112428	9.81E-04	CD180	CD180 molecule	0.193964823	down	246.07048	305.45786	57.45047	48.873547	77.37064	1.034583336	-0.27383438	0.039085228	-2.3270478	-2.5702493	-1.8993503
7905515	0.006223375	KPRP	keratinocyte proline-rich protein	0.22335493	down	964.0971	916.02783	190.62123	156.28757	386.90723	1.047330228	-0.23865764	-0.37154612	-2.534136	-2.8325756	-1.562776
7980485	0.006589807	DIO2	deiodinase, iodothyronine, type II	0.301689431	down	348.01123	391.61118	115.87063	139.93553	277.70145	0.912726293	-0.07580694	0.08176104	-1.647103	-1.3567156	-0.37040153
8008885	0.00511392	MIR21	microRNA 21	0.311376495	down	328.31882	541.65704	163.79868	166.43651	398.9946	0.993018998	-0.79659873	-0.084339775	-1.7676078	-1.7551719	-0.5485514
7906919	0.004879	RGS4	regulator of G-protein signaling 4	0.312437962	down	595.8677	562.052	170.94128	178.49202	346.11426	0.980694111	-0.003556569	-0.06483523	-1.7431935	-1.6920047	-0.75075865
8149825	7.70E-04	STC1	stanniocalcin 1	0.327394828	down	540.55115	592.4129	195.99908	190.00049	344.50354	1.015132141	-0.114629425	0.015451114	-1.5954455	-1.634569	-0.76559705
7942118	0.021816032	MYEOV	myeloma overexpressed (in a subset of t(11;14) positive multiple myelomas)	0.332834437	down	696.8137	850.91974	261.5495	209.88118	436.5808	1.087666999	-0.012605667	0.210639	-1.3764844	-1.6861945	-0.6705634
8124196	4.00E-04	DCDC2	doublecortin domain containing 2	0.334460432	down	857.3874	792.68994	265.44412	161.98586	271.14767	1.196223955	0.074899994	-0.020195326	-1.6002879	-2.3117795	-1.570478
8166202	0.001153202	GRPR	gastrin-releasing peptide receptor	0.340194281	down	741.2373	768.09766	259.56683	222.08374	330.14737	1.073708589	-0.11166731	-0.057833355	-1.6134025	-1.8325726	-1.2595329
8104901	6.56E-04	IL7R	interleukin 7 receptor	0.356825996	down	1748.4443	1956.8971	702.87775	630.3565	1271.3392	1.057831045	-0.17065716	-0.006726265	-1.4934336	-1.6436697	-0.627697
8026490	0.006938173	UCA1	urothelial cancer associated 1 (non-protein coding)	0.371830425	down	4364.5054	4598.2476	1695.2328	1796.1136	2975.9255	0.965249643	-0.17290528	-0.09010538	-1.5173887	-1.4440979	-0.7217315
8116921	2.74E-05	EDN1	endothelin 1	0.374555431	down	257.76572	309.24063	115.63662	112.28369	150.8304	1.017318495	-0.2831904	-0.01912562	-1.4358745	-1.4825116	-1.0533228
8129677	2.07E-04	SGK1	serum/glucocorticoid regulated kinase 1	0.37799282	down	349.3486	385.70908	145.50795	196.48016	343.84854	0.787793629	-0.17577522	-0.037582397	-1.4411516	-1.0102504	-0.198191
8030002	0.004676017	ZNF114	zinc finger protein 114	0.385395663	down	1367.4473	1459.849	555.9694	670.7215	799.2166	0.873044928	-0.082429886	0.03085041	-1.3447374	-1.0940889	-0.82495755
7927827	0.00803814	MYPN	myopalladin	0.388216044	down	473.06592	514.3276	194.16711	219.7082	346.13748	0.920224104	0.011569659	0.1054306	-1.2596377	-1.0797534	-0.4250803
7917875	7.07E-04	F3	coagulation factor III (thromboplastin, tissue factor)	0.39424671	down	1128.3658	1218.4924	484.61035	413.2101	943.63385	1.097291179	-0.13422585	-0.016422272	-1.3592516	-1.577437	-0.38473096
7957140	0.026575293	LGR5	leucine-rich repeat-containing G protein-coupled receptor 5	0.41232594	down	1140.3478	1191.4098	483.43637	407.03723	492.85278	1.10791244	-0.32850328	-0.28792635	-1.5660692	-1.8045807	-1.5311079
8172266	0.001339399	MIR221	microRNA 221	0.414736586	down	103.45541	157.239	65.03158	79.84388	112.838036	0.839358915	-0.49713278	0.11575731	-1.1539755	-0.86160994	-0.3640717
7931108	0.004173048	DMBT1	deleted in malignant brain tumors 1	0.428247111	down	273.21982	268.3616	113.20209	136.26653	227.5124	0.851350136	-0.059259575	-0.078999996	-1.3024846	-1.0348929	-0.29715952
7922408	0.024416802	SNORD78	small nucleolar RNA, C/D box 78	0.433920522	down	716.56525	981.90814	437.34872	597.8467	815.65576	0.705270033	-0.70518905	-0.17376582	-1.3782631	-0.92754143	-0.4915603
8178435	0.01854504	IER3	immediate early response 3	0.435939326	down	1706.7183	2088.2888	880.955	960.06036	2323.9392	0.934479296	-0.50693417	-0.2593759	-1.4571766	-1.3325319	-0.06593641
8124848	0.01894931	IER3	immediate early response 3	0.437451634	down	1474.2894	1849.911	780.84283	849.3582	2056.665	0.935911131	-0.5597636	-0.27805838	-1.470863	-1.3507309	-0.087991714
8179704	0.01887926	IER3	immediate early response 3	0.437691047	down	1473.0494	1849.291	780.9072	849.0822	2049.5693	0.936188662	-0.5608406	-0.27839026	-1.4704056	-1.35107	-0.092497826
7982377	0.003181419	GREM1	gremlin 1	0.440777703	down	1396.089	1442.4219	634.2016	727.55414	1048.3174	0.88449617	-0.12292608	-0.059360504	-1.2412373	-1.0473105	-0.5156314
8107100	4.93E-04	RGMB	RGM domain family, member B	0.445285876	down	1161.4741	1288.8168	571.5798	568.5589	717.4449	1.004211857	-0.20414543	-0.04851055	-1.2157068	-1.2298965	-0.8865544
8098214	0.001076845	TLL1	tolloid-like 1	0.44700369	down	617.0971	600.6096	266.3579	228.25226	310.12	1.114002831	0.102425575	0.062007587	-1.0996338	-1.3226457	-0.8864425
8150537	0.001323739	SLC20A2	solute carrier family 20 (phosphate transporter), member 2	0.45568695	down	729.396	753.60895	341.2662	361.7087	553.25195	0.950423525	-0.12097231	-0.0676864	-1.2015715	-1.1159967	-0.5038217
8131803	0.016932905	IL6	interleukin 6 (interferon, beta 2)	0.458081385	down	139.14098	346.5399	154.106	153.05107	274.59473	1.005482038	-1.47652	-0.18870623	-1.3150305	-1.3232375	-0.48676586
7922416	0.02741842	SNORD75	small nucleolar RNA, C/D box 75	0.462998824	down	615.6204	830.2568	380.30087	563.1294	693.7671	0.593674585	-0.5408618	-0.10278543	-1.2137051	-0.6343317	-0.35086918
8122265	4.59E-04	TNFAIP3	tumor necrosis factor, alpha-induced protein 3	0.465884664	down	280.41983	425.02457	197.28053	270.99976	489.035	0.676306655	-0.5838488	0.075600944	-1.0263543	-0.56805867	0.2836237
8121257	6.80E-04	PRDM1	PR domain containing 1, with ZNF domain	0.475909372	down	191.91118	207.01685	98.351875	96.16751	134.34299	1.020101831	-0.19519424	-0.08888308	-1.1601243	-1.1894597	-0.710676
7991335	0.002480197	ANPEP	alanyl (membrane) aminopeptidase	0.484859416	down	381.53867	468.3064	225.92558	212.13942	263.27838	1.056878098	-0.26745415	0.044560432	-0.99980116	-1.091623	-0.77440006
8109044	0.008307722	SPINK6	serine peptidase inhibitor, Kazal type 6	0.48627553	down	289.058	280.32217	134.44035	159.55878	222.22493	0.827816585	-0.035727818	-0.10530933	-1.1454635	-0.8968312	-0.41751322
7922410	0.027771054	SNORD44	small nucleolar RNA, C/D box 44	0.487291724	down	563.1214	741.8693	374.7234	533.29865	671.55945	0.568086556	-0.52353764	-0.066563606	-1.103706	-0.56162804	-0.26592922
7952335	0.02110739	SNORD14E	small nucleolar RNA, C/D box 14E	0.487425305	down	177.37115	217.07579	108.297935	162.8548	225.97215	0.498456143	-0.29724216	0.03882583	-0.99792117	-0.36867157	0.071745396
8023575	6.97E-05	CCBE1	collagen and calcium binding EGF domains 1	0.489109488	down	1334.4438	1454.0118	710.42413	474.44974	618.247	1.317345754	-0.07824961	0.047288578	-0.9844821	-1.5679289	-1.1865155
8079407	0.02648811	CCRL2	chemokine (C-C motif) receptor-like 2	0.490016958	down	111.2608	116.2006	54.570637	56.327652	65.46182	0.971490896	0.16685946	0.20278406	-0.82631236	-0.7805619	-0.56970835
7952339	0.00518261	SNORD14C	small nucleolar RNA, C/D box 14C	0.491905087	down	1350.4355	1748.2006	855.21063	1125.5211	1428.8639	0.697297306	-0.29456425	0.0909853	-0.9325628	-0.5371726	-0.20136833
7936968	0.00908412	ADAM12	ADAM metallopeptidase domain 12	0.493150144	down	570.29004	594.3409	294.17563	238.46997	271.9992	1.185583295	0.027535439	0.0953118	-0.92458934	-1.2213773	-1.0291162
8117189	0.02957003	DCDC2	doublecortin domain containing 2 /// kidney associated antigen 1	0.493563366	down	211.17659	213.08443	102.48141	95.01541	148.66173	1.067502678	-0.11151632	-0.14235528	-1.161048	-1.2576827	-0.63304186
8057803	2.21E-04	TMEFF2	transmembrane protein with EGF-like and two follistatin-like domains 2	0.49439321	down	1285.8895	1223.174	603.71826	524.0667	702.4448	1.128583133	0.11176459	0.04662927	-0.9696398	-1.1747316	-0.75080556
8136248	2.48E-04	MEST	mesoderm specific transcript homolog (mouse)	0.497507834	down	2348.255	2397.6936	1192.6979	1269.723	2118.1052	0.936078527	-0.04447524	-0.013904889	-1.0211138	-0.93070346	-0.19054508
8081341	0.011563786	FAM172B	family with sequence similarity 172, member B pseudogene	0.497895396	down	335.58542	305.1122	148.95996	162.69981	204.07649	0.912009908	0.10292848	0.004771868	-1.0013136	-0.872711	-0.55631447
8058273	0.045401264	MPP4	membrane protein, palmitoylated 4 (MAGUK p55 subfamily member 4)	0.500935096	down	224.82623	208.48872	99.4446	77.45197	89.00469	1.201685611	-0.22011025	-0.3319459	-1.3292503	-1.6982913	-1.511541
8013323	0.002845216	SNORD3A	small nucleolar RNA, C/D box 3A	0.504025882	down	810.34564	829.8828	416.96732	491.78098	679.6902	0.818816044	-0.15099971	-0.106115974	-1.0945463	-0.85315675	-0.39078045
7934979	0.006890329	ANKRD1	ankyrin repeat domain 1 (cardiac muscle)	0.504086503	down	2416.794	2424.3713	1201.4464	873.0579	1541.7865	1.268527119	-0.06829866	-0.061435383	-1.0496922	-1.5133404	-0.7052021
8013325	0.002844837	SNORD3A	small nucleolar RNA, C/D box 3A	0.504154485	down	810.44916	829.7011	416.9829	491.83835	679.7805	0.818628183	-0.150376	-0.10602156	-1.0940838	-0.8525899	-0.39017233
8013329	0.002853846	SNORD3A	small nucleolar RNA, C/D box 3A	0.504190555	down	810.69385	830.1025	417.2233	492.18442	680.0244	0.818442973	-0.1500705	-0.105361305	-1.0933203	-0.8516159	-0.38971773

8005547	0.00282709	SNORD3A	small nucleolar RNA, C/D box 3A	0.504571137	down	810.68414	829.8784	417.38794	491.90793	679.842	0.819341301	-0.15001361	-0.10583464	-1.0927051	-0.8525165	-0.39016595
8005553	0.002815761	SNORD3A	small nucleolar RNA, C/D box 3A	0.505194894	down	811.2129	830.4406	418.2028	492.50537	680.316	0.819757989	-0.14884059	-0.10444037	-1.0895284	-0.85040665	-0.38883877
8068593	0.006424058	ETS2	v-ets erythroblastosis virus E26 oncogene homolog 2 (avian)	0.506170087	down	1047.5223	1266.6337	631.59174	643.2745	912.66974	0.981603168	-0.3723558	-0.094213806	-1.0765196	-1.07326	-0.55833596
8140668	0.010206584	SEMA3A	sema domain, immunoglobulin domain (Ig), short basic domain, secreted, (semaphorin) 3A	0.506631913	down	446.10672	483.82773	246.5028	148.46509	197.91225	1.413094865	0.015639624	0.12322489	-0.8577652	-1.579267	-1.1601626
8096050	0.006982318	FGF5	fibroblast growth factor 5	0.510958945	down	1905.0526	1809.0005	912.7779	928.9057	1511.3389	0.982004694	0.22036393	0.14231174	-0.82640904	-0.80019474	-0.10482534
8004416	0.027519878	FGF11	fibroblast growth factor 11	0.511019157	down	1136.0162	1091.5507	566.7259	552.45123	742.55975	1.027198924	0.21257496	0.15693538	-0.8116153	-0.82366943	-0.39752898
8077441	0.012197754	BHLHE40	basic helix-loop-helix family, member e40	0.516682836	down	1537.2157	1545.0082	797.2336	907.9355	1703.0522	0.851958197	0.12868755	0.14553006	-0.8071191	-0.61372024	0.29642868
8015210	0.01762685	KRTAP2-4	keratin associated protein 2-4	0.516940395	down	1872.4147	1760.6198	886.9629	676.6735	929.4619	1.240700211	-0.04581483	-0.13893826	-1.0908684	-1.4849268	-1.0321249
8057797	0.006151477	SDPR	serum deprivation response	0.517501486	down	1739.6334	1421.8926	734.9427	615.2945	1172.9357	1.174173109	0.36512598	0.082235016	-0.86813	-1.1311888	-0.2024266
8159006	0.003775276	SNORD36B	small nucleolar RNA, C/D box 36B	0.518084732	down	552.83185	774.7259	403.4441	534.48804	561.9586	0.647049923	-0.41045284	0.07603836	-0.87270164	-0.46031666	-0.38519382
8019576	0.01695586	KRTAP2-4	keratin associated protein 2-4	0.519197591	down	1873.2318	1748.9916	885.8638	676.37213	928.7198	1.242712226	-0.044466972	-0.1459589	-1.0916033	-1.484499	-1.0322924
7977270	0.041151263	LOC388022	hypothetical LOC388022	0.51957515	down	951.5258	1102.7618	558.3599	484.54407	558.16064	1.135590691	-0.18726604	0.044112206	-0.9004834	-1.1048161	-0.88650733
8069676	0.010518756	ADAMTS1	ADAM metallopeptidase with thrombospondin type 1 motif, 1	0.52150885	down	970.5992	981.84686	506.2478	496.1674	745.70557	1.021195164	-0.096580505	-0.085408844	-1.0246452	-1.0559906	-0.463411
8093304	0.034125008	CCRL2	chemokine (C-C motif) receptor-like 2	0.524721244	down	107.206764	110.88019	55.811115	57.19183	67.15989	0.97492758	0.21343453	0.23321454	-0.69716233	-0.6623642	-0.4351778
7930498	0.004632095	ACSL5	acyl-CoA synthetase long-chain family member 5	0.527822584	down	312.59515	364.22678	192.81429	181.58478	242.07153	1.065511621	-0.29387394	-0.06728268	-0.9891577	-1.0650781	-0.6498809
8053735	0.012963627	LOC642838	---	0.528149887	down	324.38696	358.61575	190.53288	226.55278	266.95712	0.785701541	-0.14352655	0.032589596	-0.888391	-0.6212959	-0.38957533
8152215	0.012675474	KLF10	Kruppel-like factor 10	0.528562905	down	618.65326	618.60443	328.0775	376.77344	631.71796	0.832387517	-0.0664072	-0.06341362	-0.98326653	-0.77272195	-0.032743454
8114572	9.10E-04	HBEGF	heparin-binding EGF-like growth factor	0.528587492	down	1289.3219	1420.5739	748.2163	1037.1857	1422.2365	0.570214719	-0.09364446	0.06023534	-0.8595505	-0.3900229	0.06794453
8155083	0.016926503	CA9	carbonic anhydrase IX	0.52864866	down	2237.0488	2433.9978	1289.0945	1817.98	2936.4863	0.538052253	0.032129925	0.15056355	-0.76905537	-0.27791753	0.43701777
8140650	9.31E-04	SEMA3E	sema domain, immunoglobulin domain (Ig), short basic domain, secreted, (semaphorin) 3E	0.529798995	down	1910.5853	1847.6442	981.09814	762.4155	918.56836	1.252361242	0.077124275	0.030425072	-0.88605785	-1.2578789	-0.97583455
7954065	0.008967388	GPRC5A	G protein-coupled receptor, family C, group 5, member A	0.530719955	down	5415.0854	6017.5845	3213.7148	3484.8496	4935.184	0.903299786	-0.082829796	0.070412636	-0.8435647	-0.7198677	-0.21058305
8140534	0.004318803	SEMA3C	sema domain, immunoglobulin domain (Ig), short basic domain, secreted, (semaphorin) 3C	0.531982891	down	1040.689	1157.4467	622.152	458.20438	575.81445	1.30627544	-0.11998717	0.032942455	-0.87760574	-1.3150333	-0.9759242
7950810	0.004693448	SYTL2	synaptotagmin-like 2	0.534096251	down	289.01245	273.14795	145.48433	165.16423	206.42802	0.845845668	0.009159247	-0.07427708	-0.9791055	-0.79307145	-0.4688498
7952313	0.02200246	MIRLET7A2	microRNA let-7a-2	0.534578602	down	71.298065	90.78225	47.185688	45.571625	62.7263	1.037022713	-0.40360895	-0.08256706	-0.98609304	-1.0445625	-0.6021864
7948900	0.047873084	SNORD30	small nucleolar RNA, C/D box 30	0.536226669	down	1062.866	1342.9734	744.13226	1131.3563	1558.4254	0.353377692	-0.41125965	0.024189314	-0.87489575	-0.26690134	0.17439683
8046408	0.021873519	PDK1	pyruvate dehydrogenase kinase, isozyme 1	0.53649525	down	1335.4114	1308.2377	710.2864	1004.3118	1281.9856	0.508278684	0.12795322	0.10538801	-0.79297477	-0.27624702	0.08271917
7909271	0.020500563	IL24	interleukin 24	0.541150432	down	289.6346	286.59207	152.93205	170.86931	283.82104	0.865799362	-0.16265106	-0.16993856	-1.055837	-0.9016511	-0.17307647
8089112	1.08E-04	FILIP1L	filamin A interacting protein 1-like	0.541229892	down	423.71024	441.6539	238.83801	212.19409	290.01	1.131369983	-0.06981214	-0.009487152	-0.8951737	-1.0652889	-0.62052566
8023497	0.001651762	ATP8B1	ATPase, aminophospholipid transporter, class I, type 8B, member 1	0.541501515	down	1743.3223	1752.2147	943.55194	766.34125	1128.3184	1.219140412	0.09444078	0.100609146	-0.78435355	-1.0855879	-0.5272725
8135734	0.018630177	C7orf58	chromosome 7 open reading frame 58	0.542750975	down	445.04385	414.42285	219.90181	138.12477	148.83636	1.42040203	0.048095703	-0.079315186	-0.96095294	-1.6308423	-1.5488619
7957126	0.006106191	KCNMB4	potassium large conductance calcium-activated channel, subfamily M, beta member 4	0.546528185	down	578.618	601.63336	326.3689	375.88882	424.7438	0.820100568	-0.16580836	-0.10176531	-0.97339755	-0.76794976	-0.59142274
7948896	0.044800706	SNORD22	small nucleolar RNA, C/D box 22	0.546661315	down	486.6312	685.1773	389.4348	547.95264	647.78394	0.464000473	-0.58987397	-0.04042403	-0.91170484	-0.36773586	-0.15832679
8122396	0.021659933	AIG1	androgen-induced 1	0.550712589	down	578.8943	565.8646	306.21582	296.91663	303.06137	1.035814495	-0.11113802	-0.14227454	-1.002903	-1.0472246	-1.0260702
7938331	0.004046904	ZNF143	zinc finger protein 143	0.551967765	down	812.99805	762.4085	424.07864	506.3195	503.42856	0.756921071	0.04927508	-0.03579553	-0.89313954	-0.6340682	-0.6477521
7948332	0.037596278	LPXN	leupaxin	0.55893297	down	1123.1743	1240.499	678.24805	799.96484	998.3733	0.783518747	-0.42250443	-0.24838574	-1.0876385	-0.88441277	-0.55062866
8081235	3.22E-04	COL8A1	collagen, type VIII, alpha 1	0.561018945	down	278.6573	281.25302	157.85127	127.924805	156.77324	1.242512485	0.00386111	0.018667221	-0.8152113	-1.1296262	-0.8292063
7948902	0.005523933	SNORD29	small nucleolar RNA, C/D box 29	0.561749057	down	1252.5297	1530.7916	860.8327	1446.8434	1682.1035	0.125303507	-0.30505815	0.007483482	-0.82451886	-0.06938998	0.12068367
8086517	0.020154886	CDCP1	CUB domain containing protein 1	0.56203804	down	567.37286	588.424	322.97458	306.02682	471.2286	1.063845534	0.09568628	0.19788997	-0.6333704	-0.71641606	-0.094620705
8029489	0.010590332	BCAM	basal cell adhesion molecule (Lutheran blood group)	0.564996713	down	434.04135	490.60352	277.62723	330.04578	513.7832	0.753876124	-0.28676763	-0.11731783	-0.9410035	-0.6890208	-0.04896577
7938390	0.006285104	ADM	adrenomedullin	0.567837954	down	453.8524	525.91046	295.79016	365.15015	541.64856	0.698592475	-0.2582563	-0.05372302	-0.87017184	-0.57136375	5.12E-04
7922404	0.046049107	SNORD80	small nucleolar RNA, C/D box 80	0.569402248	down	419.56104	541.4001	310.117	464.8481	519.14014	0.3309883	-0.3835783	-0.03497982	-0.8474598	-0.23980649	-0.10152499
7912157	0.0208033	ERRFI1	ERBB receptor feedback inhibitor 1	0.570751171	down	1040.4369	1100.835	629.6761	795.8929	1120.93	0.647217107	-0.12337303	-0.04261907	-0.8516852	-0.50355786	-0.005636215
8148553	0.007190814	LY6K	lymphocyte antigen 6 complex, locus K	0.57120988	down	1369.4391	1307.546	738.1595	771.2399	947.7498	0.941901678	-0.070578255	-0.12964725	-0.93755436	-0.8903268	-0.5896943
7914342	0.03600214	FABP3	fatty acid binding protein 3, muscle and heart (mammary-derived growth inhibitor)	0.572212175	down	659.0127	638.51855	359.15756	201.39526	379.00174	1.56472559	0.10301876	0.03714466	-0.7682333	-1.5929044	-0.69216347
7958960	0.026311824	TPCN1	two pore segment channel 1	0.573019437	down	943.57886	1049.2587	587.60956	465.4668	510.6813	1.2645792	0.04046504	0.1852274	-0.6181167	-0.96149606	-0.8223693
8164200	0.01462088	ANGPTL2	angiopoietin-like 2	0.573836554	down	926.8382	963.2783	552.62665	653.9033	828.6294	0.753375763	-0.123929344	-0.07748953	-0.8787778	-0.64108914	-0.3013455
8135069	0.006763263	SERPINE1	serpin peptidase inhibitor, clade E (nexin, plasminogen activator inhibitor type 1), member 1	0.573897215	down	1688.5084	1893.5746	1091.9702	933.03546	1589.2982	1.198270793	-0.09178257	0.07461516	-0.72652054	-0.97778636	-0.17652448
7902687	0.00105719	CYR61	cysteine-rich, angiogenic inducer, 61	0.574192307	down	3191.2102	3555.2454	2034.5303	1935.9186	3078.005	1.064845611	-0.1876262	-0.03418668	-0.8345807	-0.9059207	-0.23954837
8122365	9.63E-04	GPR126	G protein-coupled receptor 126	0.576953545	down	3255.5002	3394.2551	1955.8685	1711.9575	2274.32	1.169572631	-0.1339318	-0.07432874	-0.86780167	-1.059606	-0.64950246
8086961	0.01696267	PFKFB4	6-phosphofructo-2-kinase/fructose-2,6-biphosphatase 4	0.57716613	down	523.4965	548.37933	311.2156	415.02844	603.13336	0.56227354	0.054739315	0.109812416	-0.683129	-0.2716392	0.27164236
7930413	0.00792718	DUSP5	dual specificity phosphatase 5	0.57874031	down	731.10724	777.79034	448.7023	514.44525	937.72504	0.800226863	-0.01781845	0.077947296	-0.71106464	-0.5174224	0.34741402
8095110	0.010544828	KIT	v-kit Hardy-Zuckerman 4 feline sarcoma viral oncogene homolog	0.578880986	down	851.10254	820.33936	469.07516	467.4237	547.38245	1.004701475	0.1708641	0.103894554	-0.68476677	-0.691226	-0.4689436
7954344	0.002379303	LST-3TM12	organic anion transporter LST-3b	0.579403212	down	210.61212	191.00494	110.89947	137.62312	151.48853	0.666394193	0.18014114	0.041493893	-0.7458665	-0.43159994	-0.29325423
7939173	0.01706908	DEPDC7	DEP domain containing 7	0.581429525	down	222.09216	227.36633	132.66817	158.97694	207.98027	0.722182881	-0.11697117	-0.08722973	-0.86955357	-0.59284526	-0.20927858
8146921	0.032796413	RDH10	retinol dehydrogenase 10 (all-trans)	0.581755124	down	2808.121	2933.133	1681.3607	2086.1143	3265.8738	0.676655571	0.024455706	0.07042662	-0.71108943	-0.39561877	0.24354903
8103822	0.005036932	VEGFC	vascular endothelial growth factor C	0.581950536	down	740.4969	884.1971	513.90643	421.0634	730.784	1.250730136	-0.18777275	0.06437937	-0.7166522	-1.0109158	-0.20334117
8059279	0.00245105	EPHA4	EPH receptor A4	0.582097589	down	452.9728	510.77756	295.41537	293.97098	363.19046	1.006706795	-0.057942707	0.10925356	-0.6714134	-0.67921764	-0.3850031
7901052	0.022326019	SNORD38B	small nucleolar RNA, C/D box 38B	0.582860769	down	200.06964	241.03326	140.55833	187.50291	227.75964	0.5327732	-0.4485906	-0.13036649	-0.90914327	-0.47735167	-0.21899223
8111387	0.014625718	ADAMTS12	ADAM metallopeptidase with thrombospondin type 1 motif, 12	0.583222701	down	855.9092	1071.2823	622.71436	457.1205	613.6686	1.369161158	-0.4621099	-0.13216178	-0.91004306	-1.3530852	-0.9269797
8129953	7.44E-04	HIVEP2	human immunodeficiency virus type I enhancer binding protein 2	0.586443674	down	469.15726	486.1865	284.64294	319.93665	435.1625	0.824882968	-0.11824322	-0.059521675	-0.8294573	-0.66021156	-0.21924083
7990545	0.016946977	CSPG4	chondroitin sulfate proteoglycan 4	0.589948435	down	702.3261	784.7216	460.76758	418.4305	497.57153	1.130688546	-0.01887703	0.12617429	-0.6351649	-0.7907136	-0.52331895
8145291	0.020728689	SLC25A37	solute carrier family 25, member 37	0.592937779	down	604.8725	729.2957	427.37717	395.35608	463.02805	1.106058711	-0.11400858	0.13934071	-0.6147067	-0.72616166	-0.49587092
8047097	0.04363855	GLS	glutaminase	0.593145949	down	785.49194	866.64484	509.47745	594.2702	690.7102	0.762596608	0.046697933	0.17388344	-0.57965755	-0.34378496	-0.12899208

8175666	0.015470676	GABRE	gamma-aminobutyric acid (GABA) A receptor, epsilon	0.593153654	down	268.15494	362.5793	214.17424	214.95808	285.45966	0.994718239	-0.5379793	-0.07825247	-0.8317747	-0.8254735	-0.4162334
8157216	0.005775005	UGCG	UDP-glucose ceramide glucosyltransferase	0.594124501	down	1801.9166	1969.1754	1168.6113	1052.0924	1440.6312	1.145545997	-0.0454216	0.07769998	-0.67346287	-0.8231182	-0.370718
8039484	0.022905499	IL11	interleukin 11	0.59461619	down	324.78137	314.40073	184.84926	221.92548	370.52066	0.713810889	0.20683384	0.16981728	-0.58015203	-0.31302342	0.41023096
8091411	0.028674599	TM4SF1	transmembrane 4 L six family member 1	0.596073414	down	3143.5176	2936.2961	1751.0028	2458.6865	2876.0613	0.402946342	0.23542245	0.13292058	-0.61351746	-0.1144956	0.11706066
8142975	0.00317935	MIR29A	microRNA 29a	0.596359322	down	38.086674	52.655437	31.402002	32.5679	40.251102	0.945143079	-0.54031485	-0.04311053	-0.7888568	-0.74307346	-0.4272081
7965335	0.007137872	DUSP6	dual specificity phosphatase 6	0.597546224	down	395.8148	371.2991	219.92201	300.4321	587.8361	0.46814878	0.13102691	0.044286728	-0.69859105	-0.27094015	0.6999178
8150509	0.029370645	PLAT	plasminogen activator, tissue	0.598056186	down	3176.1614	3517.9941	2077.7217	1654.484	2300.3816	1.293859481	-0.059979755	0.07535585	-0.66629124	-0.99885464	-0.52153873
7927631	0.002024163	DKK1	dickkopf homolog 1 (Xenopus laevis)	0.59916979	down	5488.148	5683.61	3412.7004	3024.569	4821.6597	1.170914509	-0.07881864	-0.030226389	-0.76918954	-0.942241	-0.2661282
8161580	0.037926808	LOC100132426	---	0.599624815	down	312.10394	324.22794	194.08154	229.83478	256.37845	0.725284449	-0.043962795	0.03283723	-0.70503074	-0.44486937	-0.29173756
7952577	0.003451568	KIRREL3	kin of IRRE like 3 (Drosophila)	0.601223924	down	564.1985	643.86523	385.90283	302.59732	371.3938	1.322936637	-0.15723579	0.03161335	-0.7024123	-1.0609833	-0.7627166
7924450	5.61E-04	DUSP10	dual specificity phosphatase 10	0.601414768	down	331.09866	353.03116	212.22156	280.01205	318.16354	0.518566277	-0.10400677	-0.011176109	-0.7447438	-0.3523682	-0.16554801
8161563	0.041482523	AQP7	---	0.603836402	down	312.91522	325.11362	196.27684	229.47581	256.55618	0.742317605	-0.04475546	0.034502346	-0.6932681	-0.449845	-0.29357228
8038949	0.024700608	ZNF841	zinc finger protein 841	0.60395095	down	621.3122	701.0173	417.38962	473.014	497.2748	0.8038824	-0.08385531	0.07567152	-0.65182525	-0.47577858	-0.40169176
8162940	0.016836245	ABCA1	ATP-binding cassette, sub-family A (ABC1), member 1	0.604498655	down	190.45872	193.34021	115.12765	132.14034	154.65564	0.782481356	-0.16081016	-0.10155264	-0.8277416	-0.63021785	-0.40334478
8160431	0.005620883	LOC554202	hypothetical LOC554202	0.605164779	down	218.05989	200.78131	120.74823	138.45271	211.49077	0.778785472	0.09350809	-0.013253212	-0.7378532	-0.54339075	0.07147074
8043484	0.037097964	LOC642838	---	0.606271	down	313.18527	324.3837	196.29436	228.73988	258.55887	0.746696173	-0.031728745	0.040736515	-0.6812288	-0.4459003	-0.27217755
8120967	0.042635027	NT5E	5'-nucleotidase, ecto (CD73)	0.606559676	down	2389.8474	2501.12	1498.274	1311.1865	1846.9701	1.18655656	0.01834774	0.092170715	-0.6291078	-0.82912034	-0.33262
8107673	0.018621244	GRAMD3	GRAM domain containing 3	0.60806512	down	1212.348	1265.4978	769.1761	833.85205	1273.2703	0.869689457	-0.09093698	-0.020461401	-0.73816365	-0.61434394	-0.010935783
7982597	0.02119457	THBS1	thrombospondin 1	0.609465115	down	4870.4653	5542.1665	3404.744	3116.0117	3786.0918	1.135084336	-0.23625469	-0.05524667	-0.7696311	-0.9001233	-0.59970635
8115831	0.002361618	DUSP1	dual specificity phosphatase 1	0.609696169	down	1339.707	1425.3507	864.7388	1061.3597	1818.1217	0.64927448	-0.120399155	-0.03785674	-0.7516944	-0.4654646	0.3180186
8123936	0.001583234	NEDD9	neural precursor cell expressed, developmentally down-regulated 9	0.610529311	down	388.07568	368.9524	225.15979	256.991	384.19363	0.778631113	0.012928963	-0.055813152	-0.76768064	-0.57703906	0.004911423
8019709	0.036144167	RNU2-1	---	0.612206566	down	4948	5719.2476	3488.0703	3556.2363	4919.779	0.969448416	-0.3654251	-0.15945752	-0.8673671	-0.843771	-0.3804995
8147344	4.36E-04	PDP1	pyruvate dehyrogenase phosphatase catalytic subunit 1	0.613579446	down	3768.7178	3709.1387	2279.9219	2042.5609	3392.7156	1.166077673	0.038540203	0.015441577	-0.68923634	-0.84556264	-0.11322149
7976567	0.014651937	BDKRB1	bradykinin receptor B1	0.613696779	down	258.311	362.4788	219.37517	230.59247	383.39536	0.921614148	-0.43887725	0.030180296	-0.6742218	-0.60294026	0.12860076
8056257	0.03747134	FAP	fibroblast activation protein, alpha	0.614901549	down	81.46807	86.70208	52.424976	51.82912	61.09967	1.117383499	0.09505526	0.19946559	-0.50210714	-0.51447886	-0.29928032
8164269	0.02301013	ENG	endoglin	0.615235179	down	1363.6178	1616.9824	987.71173	933.80304	1245.8604	1.085668525	-0.13640785	0.09705067	-0.60373944	-0.68325645	-0.2637364
8121749	0.029845696	GJA1	gap junction protein, alpha 1, 43kDa	0.615548635	down	375.6912	406.84827	247.83923	201.65218	174.50165	1.290468077	-0.15977955	-0.039022762	-0.73907804	-1.0391213	-1.2417946
7969438	3.01E-05	LMO7	LIM domain 7	0.615562655	down	1248.026	1257.4818	774.28534	664.2758	951.554	1.227670418	-0.010588328	0.00300471	-0.69701767	-0.9186004	-0.4017439
7966026	0.001316903	NUAK1	NUAK family, SNF1-like kinase, 1	0.619680584	down	470.18396	492.11026	304.75977	281.47653	391.58774	1.124276376	-0.05728213	0.031693142	-0.6587102	-0.77159244	-0.29987875
8118314	0.049473792	HSPA1B	heat shock 70kDa protein 1B	0.619881484	down	3955.8132	4084.575	2525.3633	2489.4097	2763.1335	1.023058832	-0.15915172	-0.1104606	-0.80039626	-0.8179868	-0.6703847
7948898	0.03016051	SNORD31	small nucleolar RNA, C/D box 31	0.62131224	down	470.52292	579.20087	366.4708	421.5588	520.02405	0.741042721	-0.44026026	-0.044939358	-0.73154896	-0.51969653	-0.22006543
8104568	0.002261683	LOC100133299	GALI1870	0.622567512	down	232.79295	276.60815	171.60303	142.08278	156.88322	1.28113153	-0.25547585	-0.002542178	-0.68624	-0.9861186	-0.8226183
8053602	0.018158423	PLGLB1	plasminogen-like B1	0.62305921	down	96.147095	105.14822	65.32581	81.779945	84.11411	0.586812174	-0.09718307	0.03791396	-0.6446449	-0.3177333	-0.2736988
7958600	0.029822	ANKRD13A	ankyrin repeat domain 13A	0.623482794	down	2194.7812	2237.0237	1371.7467	1551.7037	1917.9061	0.792023826	0.027038893	0.049787205	-0.6317911	-0.45362917	-0.14827602
7932132	0.022192795	FRMD4A	FERM domain containing 4A	0.624490182	down	361.98535	405.37668	249.0485	209.21584	238.71832	1.254802095	-0.092619576	0.059322994	-0.61992615	-0.8721897	-0.68663996
7921434	0.002747816	AIM2	absent in melanoma 2	0.625136475	down	80.93522	93.86108	58.93121	55.581066	67.55994	1.095910577	-0.24332666	-0.025718689	-0.70347565	-0.7833459	-0.5026641
8043367	0.018852403	PLGLB1	plasminogen-like B1	0.626224621	down	94.73276	103.58286	64.71482	80.84213	83.934395	0.585075296	-0.096143566	0.038905304	-0.6363425	-0.31219372	-0.25431824
7933672	0.006504023	PCDH15	protocadherin-related 15	0.627833412	down	472.3918	487.24088	304.10733	173.46846	186.09883	1.713353015	-0.08926996	-0.042018574	-0.7135649	-1.523407	-1.4259537
8098328	0.003183689	GALNT7	UDP-N-acetyl-alpha-D-galactosamine:polypeptide N-acetylgalactosaminyltransferase 7	0.629040722	down	1400.4434	1365.3981	859.137	743.593	883.0295	1.228235391	-0.016583124	-0.053923924	-0.7226985	-0.9310916	-0.6811981
8112342	0.006614429	ADAMTS6	ADAM metallopeptidase with thrombospondin type 1 motif, 6	0.629893207	down	402.09143	441.2307	276.781	220.63612	264.07535	1.341410656	-0.23918724	-0.09541575	-0.7622366	-1.0880874	-0.8298116
8160260	0.009517251	BNC2	basonuclin 2	0.631344499	down	375.39697	397.0118	249.3171	218.4721	260.59885	1.208842971	-0.001149496	0.073326744	-0.5901739	-0.7798287	-0.5294118
8145281	0.00963188	SLC25A37	solute carrier family 25, member 37	0.634480326	down	974.4124	1101.6354	694.11743	715.16284	849.75006	0.948357099	-0.16465442	0.011452039	-0.6449006	-0.6037753	-0.354201
8152703	0.008592828	FBXO32	F-box protein 32	0.634721917	down	247.91124	297.0961	188.40034	183.37547	225.99487	1.046228758	-0.3606	-0.09747251	-0.7532759	-0.78986263	-0.48647484
8012883	0.020409627	HS3ST3A1	heparan sulfate (glucosamine) 3-O-sulfotransferase 3A1	0.638398264	down	661.71045	650.5556	411.26956	432.2427	513.92163	0.912351176	-0.0874602	-0.10496775	-0.7524392	-0.6888978	-0.44007936
7941694	0.004769899	RBM14	RNA binding motif protein 14	0.638411469	down	1215.6621	1324.4819	848.85547	1019.6914	1334.4406	0.640819098	-0.081041336	0.042009037	-0.6054325	-0.33479372	0.054293316
8083594	0.02929325	PTX3	pentraxin 3, long	0.640499282	down	257.5906	328.72122	207.82695	179.22675	224.32239	1.236572006	-0.2957295	0.0787309	-0.5640003	-0.7768666	-0.45358673
7953532	7.23E-04	ENO2	enolase 2 (gamma, neuronal)	0.641763134	down	2423.3435	2473.842	1584.2355	1868.7535	2311.92	0.680175448	-0.00322024	0.030428886	-0.60945827	-0.37111315	-0.066316605
8068022	0.006783839	MIR155	microRNA 155	0.64191939	down	87.727974	77.381035	49.58256	71.866974	88.16175	0.198358399	0.11931753	-0.06661526	-0.7061512	-0.16671865	0.12287712
8105040	0.002624022	OSMR	oncostatin M receptor	0.642295905	down	3239.1726	3456.721	2225.4211	2030.2218	2722.1638	1.158531078	-0.11962795	-0.02713426	-0.66582423	-0.8024349	-0.37034068
8136200	0.036489204	CPA4	carboxypeptidase A4	0.642305971	down	3137.9573	3288.0293	2109.8787	1783.014	2444.1995	1.27743881	-0.13066673	-0.057943344	-0.69661075	-0.9383898	-0.47672653
7965040	0.005518349	PHLDA1	pleckstrin homology-like domain, family A, member 1	0.642523091	down	533.58014	528.3749	339.53928	324.27716	538.19244	1.080822252	-0.011616389	-0.01884079	-0.65702057	-0.72368526	0.002264659
8147721	0.027266918	FLJ45248	FLJ45248 protein	0.642543939	down	228.5304	211.95448	137.44225	142.0218	204.39555	0.93853962	0.1470248	0.034908455	-0.60322446	-0.5392373	-0.019206205
8147548	1.06E-05	POP1	processing of precursor 1, ribonuclease P/MRP subunit (S. cerevisiae)	0.643259028	down	218.89183	239.52301	154.08205	154.29497	169.19777	0.997507987	-0.13937044	-0.005551974	-0.6420803	-0.6463701	-0.5092001
7915472	0.00815958	SLC2A1	solute carrier family 2 (facilitated glucose transporter), member 1	0.644311174	down	5890.7676	6026.519	3899.513	4672.8022	5926.863	0.636442398	-0.092450775	-0.06074174	-0.69491225	-0.43639565	-0.083517395
8077851	6.98E-05	HRH1	histamine receptor H1	0.644641335	down	238.56299	246.6947	158.95364	182.48616	215.76033	0.731795809	-0.07464107	-0.019890467	-0.6533219	-0.4641684	-0.213823
8046922	0.036339954	COL3A1	collagen, type III, alpha 1	0.644717766	down	484.56802	525.9654	334.38214	276.59933	336.781	1.301606779	0.029040972	0.14462757	-0.48863283	-0.7712124	-0.48044267
8105302	2.04E-04	FST	follistatin	0.645725154	down	508.6049	603.23566	389.41382	389.72095	489.2797	0.998563617	-0.27795696	-0.021766344	-0.65277416	-0.6626215	-0.3282194
8038942	0.034797143	ZNF432	zinc finger protein 432	0.646756066	down	278.55066	292.71793	188.64946	242.92175	274.0376	0.478494399	-0.049616497	0.009378433	-0.619328	-0.24777906	-0.07396253
8108370	0.01206518	EGR1	early growth response 1	0.647182336	down	1641.3634	1659.3114	1083.2362	2742.319	4058.5527	-1.879976086	-0.053749084	-0.033155758	-0.66091156	0.69052666	1.2546221
7952116	0.019977083	BCL9L	B-cell CLL/lymphoma 9-like	0.647933904	down	767.2173	755.5317	489.9236	467.02725	621.33545	1.086203508	-0.04404831	-0.053913753	-0.67999524	-0.749677	-0.32997417
8112615	0.007913281	ENC1	ectodermal-neural cortex 1 (with BTB-like domain)	0.648186355	down	348.1051	327.25473	210.95885	202.28339	281.99182	1.074598171	0.15763378	0.08167521	-0.5438442	-0.60454434	-0.12728913
8041048	0.005378059	FOSL2	FOS-like antigen 2	0.649242175	down	1029.1572	1111.3053	717.81256	867.2742	1371.6416	0.620166715	-0.14210765	-0.032046	-0.65521747	-0.38299623	0.27970156
8010139	9.53E-05	SEC14L1	SEC14-like 1 (S. cerevisiae)	0.649454646	down	1110.0829	1228.8755	798.6223	719.0561	915.3543	1.184928781	-0.15640385	-0.008296649	-0.63099605	-0.78510886	-0.43844858

8067409	0.016173175	LAMA5	laminin, alpha 5	0.649728969	down	484.6246	519.86053	341.20038	300.4476	386.52896	1.228102238	-0.13171673	-0.028068542	-0.6501586	-0.8216184	-0.45586967
7915392	0.004830344	HIVEP3	human immunodeficiency virus type I enhancer binding protein 3	0.649738636	down	299.29446	299.2153	195.03621	187.0293	216.63025	1.07685717	0.0237662	0.02772522	-0.59434336	-0.64895374	-0.44627428
8152222	0.041147813	AZIN1	antizyme inhibitor 1	0.650490063	down	1017.95026	947.06396	623.95636	693.38324	1056.6136	0.785127679	0.11260033	0.004339536	-0.6160615	-0.44428888	0.16452599
8155864	0.0263264	RORB	RAR-related orphan receptor B	0.650699245	down	419.44937	404.6451	259.36612	243.95764	303.26526	1.106061317	0.20355415	0.1379401	-0.481997	-0.57409525	-0.25685373
8005707	0.017607983	MAP2K3	mitogen-activated protein kinase kinase 3	0.651571705	down	1052.4768	1200.6724	780.6473	775.0796	1129.148	1.013255636	-0.20482826	-0.007492701	-0.62549686	-0.63365716	-0.088661514
7988077	0.013438887	LCMT2	leucine carboxyl methyltransferase 2	0.653762699	down	723.33	697.2534	452.07242	510.8488	757.11865	0.760273493	0.045062702	-0.009496689	-0.6226578	-0.45609665	0.11811002
7921900	0.024884325	SH2D1B	SH2 domain containing 1B	0.653936913	down	177.4207	157.72783	101.66666	101.24347	130.61548	1.007548719	0.1407218	-0.035339672	-0.6481163	-0.6618166	-0.31569862
8116910	0.009038708	HIVEP1	human immunodeficiency virus type I enhancer binding protein 1	0.654993461	down	552.19525	568.7196	371.94446	345.21194	405.3793	1.135853137	-0.12771957	-0.07967472	-0.6901223	-0.79695606	-0.5629015
8132250	0.039894495	BMPER	BMP binding endothelial regulator	0.656354851	down	358.3444	383.76038	247.9394	178.07422	191.8289	1.514391665	-0.099230446	-0.017450333	-0.6249024	-1.1049153	-1.0022802
8066214	0.044211168	TGM2	transglutaminase 2 (C polypeptide, protein-glutamine-gamma-glutamyltransferase)	0.657144641	down	478.92905	509.64798	331.66306	290.11942	358.95422	1.23341101	-0.018958092	0.10067908	-0.50503796	-0.698322	-0.41660595
8109149	0.002034455	PCYOX1L	prenylcysteine oxidase 1 like	0.658754064	down	929.53265	902.3499	593.56323	627.33154	840.9639	0.890641944	0.046348255	0.008061727	-0.5941264	-0.5137612	-0.091752686
8122202	0.007345939	MYB	v-myb myeloblastosis viral oncogene homolog (avian)	0.659400121	down	214.78465	212.09839	139.52846	191.16652	207.89885	0.288437236	-0.031978767	-0.04507065	-0.64584464	-0.18858941	-0.06803862
8153021	0.03713922	ST3GAL1	ST3 beta-galactoside alpha-2,3-sialyltransferase 1	0.660400304	down	490.24612	525.66766	342.90775	338.93222	386.07715	1.021752747	0.023484865	0.12692769	-0.47165966	-0.48924288	-0.29883417
8078918	0.035044808	SNORA62	small nucleolar RNA, H/ACA box 62	0.661257055	down	364.19046	477.61423	314.88712	409.47348	473.50455	0.418742458	-0.3111798	0.07391516	-0.5228017	-0.13689995	0.061451275
8037374	0.021275803	PLAUR	plasminogen activator, urokinase receptor	0.661458169	down	2339.031	2475.583	1638.0083	1624.2438	2182.1572	1.016433758	-0.13565762	-0.053407032	-0.6496852	-0.6671095	-0.22922389
8129937	0.004113168	CITED2	Cbp/p300-interacting transactivator, with Glu/Asp-rich carboxy-terminal domain, 2	0.661511639	down	3295.297	3302.7683	2179.6345	2186.8845	3470.214	0.993544847	0.014200528	0.017157555	-0.579004	-0.58097076	0.085720696
8025601	0.002444303	ICAM1	intercellular adhesion molecule 1	0.662759391	down	796.73	1066.2761	704.3786	795.0153	1110.2345	0.749551461	-0.45818648	-0.022716522	-0.61615944	-0.44675922	0.036410015
8162759	0.004671053	TBC1D2	TBC1 domain family, member 2	0.664187829	down	1333.6609	1379.3358	915.36816	795.3933	1045.335	1.258584543	-0.10582733	-0.057202976	-0.6475398	-0.8555104	-0.46099472
8083569	0.007560857	TIPARP	TCDD-inducible poly(ADP-ribose) polymerase	0.665577427	down	800.02246	718.41437	480.88696	556.6777	693.9646	0.68091792	0.17272885	0.026387215	-0.56093436	-0.3429664	-0.023075739
8046726	6.79E-04	SSFA2	sperm specific antigen 2	0.665794831	down	1816.4481	1829.3256	1216.1737	1334.9352	1563.3326	0.806309823	0.03609848	0.045202572	-0.5416479	-0.40716457	-0.17960358
8026047	0.030289765	JUNB	jun B proto-oncogene	0.665986428	down	514.21783	583.78015	385.43176	506.51953	776.689	0.389519774	-0.2766069	-0.092477165	-0.67891246	-0.2819821	0.33507505
8085914	0.011053873	SLC4A7	solute carrier family 4, sodium bicarbonate cotransporter, member 7	0.666183817	down	908.22675	933.9741	621.5363	625.6576	746.6646	0.986809215	-0.095666885	-0.06187439	-0.64788216	-0.63358563	-0.37973595
7920642	0.007112539	MUC1	mucin 1, cell surface associated	0.666460908	down	237.69295	262.76578	174.0144	176.91096	240.38835	0.967363212	-0.056384247	0.10218763	-0.48322025	-0.46359715	-0.028462887
8127502	0.04578795	C6orf155	chromosome 6 open reading frame 155	0.666490713	down	253.39986	325.00436	215.21532	247.57234	271.5355	0.705280053	-0.31392765	0.03699525	-0.5483481	-0.3438274	-0.20739634
8005048	0.001123286	MYOCD	myocardin	0.666554685	down	60.979584	68.44672	45.60841	51.9073	56.261265	0.724196318	-0.18224399	-0.016976833	-0.60218173	-0.414598	-0.298594
7956426	1.36E-04	INHBE	inhibin, beta E	8.135448	up	110.78947	112.143745	893.12787	328.81845	87.88285	0.27743804	0.16475455	0.18552525	3.209747	1.7646679	-0.14547174
7954398	2.81E-04	C12orf39	chromosome 12 open reading frame 39	7.936671	up	86.517296	80.11077	654.7015	319.47266	86.78193	0.416578057	0.114854656	0.007533868	2.9960678	1.9908687	0.12313318
7940582	6.39E-05	BEST1	bestrophin 1	7.259951	up	107.290306	110.00526	796.24115	381.65372	92.619545	0.39585289	-0.097455345	-0.06267961	2.7972803	1.7241911	-0.3110682
7915543	8.92E-06	SLC6A9	solute carrier family 6 (neurotransmitter transporter, glycine), member 9	5.264593	up	234.17644	274.5486	1443.3065	789.4116	224.01521	0.440521514	-0.29350868	-0.0655907	2.3307314	1.4583601	-0.3619868
7964460	7.66E-04	DDIT3	DNA-damage-inducible transcript 3	4.384938	up	417.9269	422.03207	1854.5094	1477.0352	614.8411	0.736488535	0.006955147	0.02014192	2.1526983	1.8408579	0.5798149
8082916	2.58E-04	IL20RB	interleukin 20 receptor beta	4.0784883	up	94.1616	92.86446	383.3134	156.43648	102.68864	0.218875028	-0.001752218	-0.01950852	2.0085258	0.72353536	0.12613392
7981290	3.29E-04	WARS	tryptophanyl-tRNA synthetase	3.9474723	up	968.5322	977.06586	3834.071	2897.501	1235.3126	0.672184699	-0.05668831	-0.049841244	1.9310879	1.5323397	0.3028113
7965979	1.23E-04	ALDH1L2	aldehyde dehydrogenase 1 family, member L2	3.9092891	up	424.14377	388.99158	1507.8383	862.9092	256.79166	0.423576895	0.16036065	0.02861754	1.9955238	1.1849076	-0.56336325
8102800	0.003536172	SLC7A11	solute carrier family 7, (cationic amino acid transporter, y+ system) member 11	3.642926	up	1320.6085	1292.1302	4535.8013	3125.7686	1065.652	0.565297265	0.016830444	-0.028644243	1.8364534	1.3034706	-0.255222
8141150	8.81E-04	ASNS	asparagine synthetase (glutamine-hydrolyzing)	3.479328	up	417.16806	363.2513	1261.5853	1048.7379	373.82153	0.763064295	0.230388	0.022573471	1.8213822	1.5641598	0.075330414
8006531	0.001908512	SLFN5	schlafen family member 5	3.0873232	up	337.89438	290.01025	915.1795	530.3796	357.77628	0.384486841	0.21122074	-0.010301908	1.6160545	0.85377216	0.28797245
8000574	6.02E-04	NUPR1	nuclear protein, transcriptional regulator, 1	2.9977584	up	144.42157	143.11455	428.4447	322.72668	170.4084	0.629488787	-0.10403665	-0.11715126	1.4667329	1.0612403	0.1424826
7982868	5.73E-04	CHAC1	ChaC, cation transport regulator homolog 1 (E. coli)	2.9877472	up	283.88522	283.00122	836.3957	636.4451	435.4691	0.638683422	0.122963585	0.129018	1.708076	1.3087331	0.76750135
8060344	3.35E-04	TRIB3	tribbles homolog 3 (Drosophila)	2.9265392	up	347.01123	333.50507	974.28217	637.9331	382.6785	0.475091931	-0.02376461	-0.08418751	1.4650081	0.85755634	0.11586412
7995895	9.34E-06	HERPUD1	homocysteine-inducible, endoplasmic reticulum stress-inducible, ubiquitin-like domain member 1	2.9262369	up	1435.4406	1492.9722	4366.016	3713.4075	1365.7559	0.772851183	-0.072979294	-0.015663147	1.5333834	1.3000555	-0.1466217
8058450	0.002552386	GPR1	G protein-coupled receptor 1	2.8386288	up	96.14231	86.60803	249.30293	149.61983	93.096306	0.387300401	0.14374685	-0.002611637	1.5025826	0.78920716	0.108025394
7973530	1.93E-04	PCK2	phosphoenolpyruvate carboxykinase 2 (mitochondrial)	2.7423804	up	559.6592	528.23724	1444.8608	1005.3794	491.4409	0.520543199	0.178243	0.08982881	1.5452576	1.0226902	-0.010420799
8003060	0.00838013	SDR42E1	short chain dehydrogenase/reductase family 42E, member 1	2.7128682	up	150.80492	130.53175	341.20828	432.29025	357.59152	1.432330882	0.078980766	-0.11343781	1.3263812	1.6689277	1.3951465
7925342	3.34E-04	ERO1LB	ERO1-like beta (S. cerevisiae)	2.699252	up	124.00741	123.233315	333.079	351.70413	167.97972	1.088756316	8.34E-05	-0.013180415	1.4193792	1.4996761	0.43644205
8122724	0.001957933	ULBP1	UL16 binding protein 1	2.695981	up	328.51794	303.01123	826.75995	788.8921	479.49585	0.927698439	0.083875656	-0.029457092	1.4013532	1.3415855	0.6332369
8063386	3.03E-04	CEBPB	CCAAT/enhancer binding protein (C/EBP), beta	2.644258	up	471.52426	541.2747	1437.6963	1053.1722	681.1717	0.571045477	-0.16798179	0.042170525	1.4450334	0.9990613	0.37187162
7904433	3.88E-04	PHGDH	phosphoglycerate dehydrogenase	2.571933	up	2215.3013	1929.3485	4938.7676	4341.1953	2170.7874	0.801432675	0.1560653	-0.048242886	1.3146101	1.1291491	0.12755997
8156043	3.01E-04	PSAT1	phosphoserine aminotransferase 1	2.5668552	up	1234.7496	1111.1805	2848.2146	2141.3127	1073.194	0.593040862	0.21128781	0.059978485	1.4199804	1.0121197	0.01345094
8078991	0.016611537	ZNF619	zinc finger protein 619	2.5580332	up	97.653564	99.77322	256.04764	317.49197	290.7941	1.393182262	-0.23838012	-0.20824115	1.1467938	1.4956633	1.3594409
7922846	9.69E-04	FAM129A	family with sequence similarity 129, member A	2.522511	up	496.85318	473.8689	1193.7448	790.41986	468.95218	0.439729903	0.056846935	-0.019654592	1.3152059	0.7275689	-0.027927399
8144874	0.002826294	NSAP11	nervous system abundant protein 11	2.4826944	up	127.6645	97.89689	246.04558	201.86528	127.23522	0.701784066	0.37538257	-0.010275205	1.3016315	1.0374217	0.36655998
8002347	8.37E-06	AARS	alanyl-tRNA synthetase	2.4782848	up	2188.205	2037.8711	5047.2036	4123.003	2160.1355	0.692888506	0.13565095	0.036165554	1.3455076	1.0526505	0.11653391
7989037	0.003604754	CCPG1	cell cycle progression 1	2.4698458	up	120.365326	112.88403	279.32132	237.86668	162.23535	0.750929374	0.21392171	0.109735966	1.4141569	1.1944065	0.6391673
7969651	3.93E-04	DNAJC3	DnaJ (Hsp40) homolog, subfamily C, member 3	2.3997738	up	531.0598	516.1541	1243.8241	1100.8463	489.16907	0.803512856	0.03797245	-0.00440979	1.2584887	1.0879167	-0.080916084
8151559	0.006520338	SLC10A5	solute carrier family 10 (sodium/bile acid cotransporter family), member 5	2.361314	up	101.105095	100.40293	235.21008	281.88004	145.1568	1.346197958	-0.00569582	-0.038239162	1.2013508	1.4602841	0.5176771
7899436	0.002574881	SESN2	sestrin 2	2.3513336	up	290.23535	275.56372	646.11414	619.6753	439.7931	0.928649818	-0.002083937	-0.07860216	1.1548771	1.0997034	0.59842366
8100097	0.014308128	GABRG1	gamma-aminobutyric acid (GABA) A receptor, gamma 1	2.3430867	up	99.48474	87.31571	204.37111	122.4165	67.20046	0.299864765	0.010058244	-0.16827536	1.060135	0.32700872	-0.51704246
7906900	1.11E-04	DDR2	discoidin domain receptor tyrosine kinase 2	2.220915	up	416.1411	398.20438	883.5629	606.6591	334.8851	0.429486063	-0.00894324	-0.06162262	1.0895315	0.54164535	-0.31381035
8017850	3.87E-04	WIPI1	WD repeat domain, phosphoinositide interacting 1	2.2058547	up	726.0945	731.23267	1613.2456	1215.466	626.5721	0.549009333	0.004697482	0.02068297	1.1620207	0.75507164	-0.20002396
7940717	0.001087838	SLC3A2	solute carrier family 3 (activators of dibasic and neutral amino acid transport), member 2	2.1986167	up	2756.4246	3074.0938	6705.5117	6710.6987	3361.416	1.001428368	-0.1258173	0.027749697	1.1643457	1.1600447	0.16681194
8014100	0.023762202	C17orf75	chromosome 17 open reading frame 75	2.160512	up	348.73462	325.68933	685.5869	777.8206	648.533	1.25627764	-0.17818372	-0.27279663	0.8385766	1.0290931	0.7693958
7902290	0.009820903	CTH	cystathionase (cystathionine gamma-lyase)	2.1542776	up	262.80963	237.52632	512.561	443.21948	242.752	0.747880813	0.29905924	0.13966973	1.2468739	1.0527906	0.18694925
8071559	0.001830122	SDF2L1	stromal cell-derived factor 2-like 1	2.152995	up	296.79538	284.44363	610.8337	517.0994	275.3184	0.712815099	0.07698027	0.006120841	1.1124659	0.87488365	-0.03683694

7952145	3.05E-04	HYOU1	hypoxia up-regulated 1	2.1372762	up	1493.7938	1479.6927	3166.6672	2670.3643	1392.6215	0.705802963	0.05802886	0.0456775	1.1414509	0.89784116	-0.040647507
8042310	5.77E-05	SLC1A4	solute carrier family 1 (glutamate/neutral amino acid transporter), member 4	2.1366994	up	503.51624	495.51004	1057.8104	868.0915	489.60925	0.662602208	0.018739065	-0.005352338	1.0900316	0.80203754	-0.024966558
8020955	7.83E-04	MOCOS	molybdenum cofactor sulfurase	2.1353278	up	650.87134	611.50214	1301.8958	903.4279	518.66864	0.422839572	0.040871304	-0.051081657	1.043376	0.5179561	-0.28422198
7928308	0.025387567	DDIT4	DNA-damage-inducible transcript 4	2.1305234	up	297.29672	239.98541	506.91653	436.00797	436.14584	0.734356339	0.52161187	0.19319518	1.2844032	1.0862843	1.092501
8027566	8.32E-05	CEBPG	CCAAT/enhancer binding protein (C/EBP), gamma	2.1145322	up	577.627	546.0611	1152.9513	1034.1185	642.0491	0.804193905	0.055111885	-0.01927185	1.0610666	0.9029109	0.21583748
8143684	3.64E-05	PDIA4	protein disulfide isomerase family A, member 4	2.1092854	up	2789.8337	2860.128	6040.235	4760.2373	2337.8918	0.597498543	-0.034074146	0.002252579	1.0790068	0.7315197	-0.28868675
7972840	0.043536887	TUBGCP3	tubulin, gamma complex associated protein 3	2.0748003	up	454.688	410.60913	806.15234	905.1873	780.5158	1.250377095	-0.048116367	-0.20058663	0.8523858	1.0215029	0.8075908
7908147	0.008749443	TSEN15	tRNA splicing endonuclease 15 homolog (S. cerevisiae)	2.0641222	up	597.0811	555.2226	1131.1313	832.7149	682.13727	0.481833839	-0.021001497	-0.13722165	0.9083068	0.467508	0.18454552
7991401	4.61E-04	HDDC3	HD domain containing 3	2.0633333	up	343.72476	322.69388	666.58655	624.96466	526.4859	0.878968371	0.096338905	0.011781693	1.0567585	0.96769714	0.7160044
8016578	0.010580584	SLC35B1	solute carrier family 35, member B1	2.0565665	up	994.0161	967.81934	1969.9242	2000.8527	1237.569	1.030863537	0.09807142	0.054139137	1.0943769	1.1229671	0.42445755
8070632	0.001467338	CBS	cystathionine-beta-synthase	2.04978	up	310.37604	297.35187	606.6895	406.24063	262.7347	0.352006188	0.0586284	-0.007723808	1.0277452	0.4414495	-0.18223412
8059642	0.007906356	SLC16A14	solute carrier family 16, member 14 (monocarboxylic acid transporter 14)	2.0486631	up	196.3868	185.21968	373.5579	518.3869	370.95578	1.768983587	-0.013663451	-0.103233814	0.93144894	1.4060723	0.92099315
8042830	0.002242392	MTHFD2	methylenetetrahydrofolate dehydrogenase (NADP+ dependent) 2	2.0475454	up	1591.3456	1509.4956	3124.4954	3004.9268	1754.4375	0.925963706	0.067661285	-0.001799266	1.0320963	0.9884799	0.2109232
8124691	0.00178294	HCG8	HLA complex group 8	2.0264323	up	188.65587	183.6791	373.98282	311.4845	202.54256	0.671586451	0.021018505	-0.013738632	1.0052034	0.69485027	0.11545452
7906878	5.63E-05	DDR2	discoidin domain receptor tyrosine kinase 2	2.0140543	up	1588.8091	1554.8634	3128.307	2320.3564	1210.7814	0.486508064	0.026777586	-0.006105741	1.0039968	0.57304126	-0.3673016
7919780	0.014615362	GOLPH3L	golgi phosphoprotein 3-like	2.0121403	up	487.25244	470.85788	940.31476	933.986	754.37134	0.986518975	-0.036946934	-0.101431526	0.90729934	0.90726185	0.60333663
8027002	0.002793437	GDF15	growth differentiation factor 15	1.9986827	up	813.7842	735.9273	1455.82	1601.661	2276.9539	1.202587136	0.28728613	0.14125855	1.140308	1.2682997	1.772262
8102532	0.03677799	PDE5A	phosphodiesterase 5A, cGMP-specific	1.9983747	up	81.85184	72.18064	149.03873	88.18833	73.2571	0.208275928	0.16315286	-0.014070828	0.9847563	0.27625242	-0.003092766
8084064	0.001031468	MTHFD2	methylenetetrahydrofolate dehydrogenase (NADP+ dependent) 2	1.995168	up	1247.8296	1202.9463	2403.1023	2197.486	1243.3597	0.828675356	0.10710684	0.054047268	1.0505575	0.92504597	0.10368061
8094625	0.006956251	KLHL5	kelch-like 5 (Drosophila)	1.9949982	up	478.54745	504.10397	1014.9265	798.83954	585.43036	0.576982323	-0.058968227	0.009268761	1.0056562	0.67336875	0.22934723
8070083	0.015638907	TMEM50B	transmembrane protein 50B	1.9906577	up	368.85718	366.9384	721.7793	703.9372	349.15237	0.949718029	-0.06990973	-0.09671179	0.8965333	0.8655965	-0.15446663
7934936	1.41E-04	SLC16A12	solute carrier family 16, member 12 (monocarboxylic acid transporter 12)	1.970452	up	85.858284	74.93578	147.89522	94.77869	71.21777	0.271971797	0.16463232	-0.024126211	0.95440036	0.31002283	-0.09997209
8111952	0.015257537	C5orf28	chromosome 5 open reading frame 28	1.9676847	up	125.02191	108.332214	218.55394	211.94318	159.4434	0.940023077	0.21862109	0.0244627	1.0009618	0.9918208	0.5810668
8126629	6.22E-04	GTPBP2	GTP binding protein 2	1.9405909	up	1141.8263	1311.2744	2539.7722	1617.1771	1155.5826	0.249005493	-0.17824428	0.01759561	0.97409153	0.32288107	-0.16212814
7949836	1.72E-04	CDK2AP2	cyclin-dependent kinase 2 associated protein 2	1.9315867	up	634.68146	666.65106	1286.1741	1154.7474	652.974	0.787858253	-0.11316713	-0.044036865	0.9057496	0.74695367	-0.072665215
7958262	0.001090017	TCP11L2	t-complex 11 (mouse)-like 2	1.9236261	up	184.23431	187.93938	359.6132	232.4652	145.66898	0.259362901	-0.098145805	-0.055423576	0.88840485	0.25202766	-0.44724464
8132070	0.00208437	GARS	glycyl-tRNA synthetase	1.9197464	up	2155.8486	2119.0237	4050.2273	3273.9636	2071.8318	0.598041501	0.14261246	0.11270332	1.053619	0.74870396	0.08833027
8053648	0.017803924	KRCC1	lysine-rich coiled-coil 1	1.9180793	up	111.54421	111.70746	218.17639	169.6159	129.05824	0.543899896	-0.011764686	-0.00799052	0.9316718	0.6019583	0.20297988
7952069	0.00240829	IFT46	intraflagellar transport 46 homolog (Chlamydomonas)	1.9170656	up	853.78546	747.7027	1440.1168	1478.5597	1372.169	1.0555201	0.19474728	0.005037626	0.9439373	0.989494	0.879886
8109576	0.001807634	THG1L	tRNA-histidine guanylyltransferase 1-like (S. cerevisiae)	1.9089563	up	386.6914	332.08408	632.1569	756.52606	698.0908	1.414463263	0.22252433	0.001196861	0.93398094	1.1951504	1.0798382
8091637	2.31E-04	SLC33A1	solute carrier family 33 (acetyl-CoA transporter), member 1	1.9073111	up	553.3466	587.5953	1121.1636	944.7955	533.47064	0.669455438	-0.109716415	-0.021909714	0.9096305	0.6638425	-0.16523616
8080781	0.02150105	PXK	PX domain containing serine/threonine kinase	1.900457	up	743.3056	749.5933	1388.0013	1252.2845	997.0086	0.787413692	-0.11929544	-0.120947205	0.80539924	0.65826863	0.32668272
8124498	0.047415383	ZNF204P	zinc finger protein 204, pseudogene	1.8946275	up	77.11196	63.794483	118.68064	149.66522	100.17014	1.564524494	0.068769455	-0.21467717	0.70723706	1.0573331	0.43901286
8003298	0.00210989	SLC7A5	solute carrier family 7 (cationic amino acid transporter, y+ system), member 5	1.8932068	up	2201.7625	2396.3953	4513.3228	3520.4004	2109.32	0.530960602	-0.22151756	-0.105337776	0.81549424	0.45112863	-0.28049693
7960320	0.032722756	DCP1B	DCP1 decapping enzyme homolog B (S. cerevisiae)	1.8682678	up	634.7399	590.48834	1066.1377	1077.0836	844.04956	1.02301254	0.11821429	0.010766983	0.91246825	0.92835397	0.576657
8135480	0.04820614	DNAJB9	DnaJ (Hsp40) homolog, subfamily B, member 9	1.8631966	up	220.43982	222.76299	431.69626	423.84854	218.03674	0.962439108	-0.08323654	-0.069449425	0.8283305	0.8574786	-0.0978748
7980547	1.12E-04	SEL1L	sel-1 suppressor of lin-12-like (C. elegans)	1.859678	up	1612.3724	1634.3052	3035.1592	2510.3616	1344.6046	0.625373094	0.031093916	0.04875374	0.94380665	0.6696367	-0.23225658
8128977	0.029683398	TUBE1	tubulin, epsilon 1	1.851697	up	156.552	143.34927	269.48383	275.73862	201.51178	1.049588233	0.06208992	-0.061074894	0.8277731	0.8939583	0.43969712
8146717	0.009511877	SGK3	serum/glucocorticoid regulated kinase family, member 3	1.8452647	up	515.43677	429.9716	802.16595	770.93634	438.57825	0.916093272	0.190382	-0.066981636	0.8168462	0.7661724	-0.038475353
7940669	0.00213173	C11orf83	chromosome 11 open reading frame 83	1.8375247	up	171.02576	172.36366	317.60413	360.16122	330.3812	1.293011239	0.009878318	0.033490974	0.9112546	1.0983683	0.97523594
8164165	6.25E-05	HSPA5	heat shock 70kDa protein 5 (glucose-regulated protein, 78kDa)	1.8302566	up	6610.6055	6816.145	12469.794	11963.3545	5152.6177	0.910422543	-0.028258959	0.016888937	0.8889348	0.8296254	-0.3861815
8117675	0.04270834	PGBD1	piggyBac transposable element derived 1	1.8252828	up	117.25204	126.8302	223.42769	383.74936	329.20633	2.659687741	-0.231939	-0.1446832	0.72343683	1.501418	1.2838174
8113733	0.005871791	CEP120	centrosomal protein 120kDa	1.8241874	up	485.03342	451.15527	815.3934	816.6844	728.7796	1.003544385	0.07639948	-0.042194683	0.82505924	0.82952243	0.6641944
8121031	0.0454485	SLC35A1	solute carrier family 35 (CMP-sialic acid transporter), member A1	1.8228612	up	166.13106	157.40471	284.00772	289.20172	250.80444	1.041025881	-0.16526985	-0.17902279	0.68718195	0.7347266	0.51115006
7956785	9.95E-04	XPOT	exportin, tRNA (nuclear export receptor for tRNAs)	1.8088498	up	1555.559	1388.7848	2515.177	2282.2175	1380.8672	0.79318083	0.108716644	-0.04629453	0.8087781	0.6719163	-0.053326923
8117667	0.0130068	ZNF187	zinc finger protein 187	1.8053242	up	112.92312	110.238945	199.32086	250.52759	211.02968	1.57482745	-0.1187884	-0.113150276	0.7391076	1.0821161	0.833296
7906786	0.007883937	FCRLA	Fc receptor-like A	1.801271	up	91.00763	95.97486	174.72963	113.29623	90.22726	0.219940583	-0.0854435	-0.005344391	0.84367085	0.22911882	-0.120749
8154381	5.64E-04	C9orf150	chromosome 9 open reading frame 150	1.8010503	up	392.37988	382.76254	689.50195	402.28784	330.2346	0.063654357	0.014876683	-0.015235901	0.8336026	0.055059116	-0.22818883
8044450	0.00217428	ZC3H6	zinc finger CCCH-type containing 6	1.7954413	up	159.11076	120.41143	216.732	189.99109	173.2644	0.722375916	0.34781346	-0.031549294	0.81278914	0.62848425	0.49422345
7945803	5.40E-06	CARS	cysteinyl-tRNA synthetase	1.7843045	up	540.7121	500.73746	893.4655	740.07294	518.96216	0.609417856	0.120407425	0.012316068	0.8476779	0.57413226	0.063689865
8080084	4.81E-04	MANF	mesencephalic astrocyte-derived neurotrophic factor	1.7748735	up	2530.1052	2497.4607	4420.5127	3963.0706	1934.0884	0.762127025	-0.048445065	-0.07000414	0.75771207	0.59770554	-0.43478966
8044021	0.024556696	IL1RL1	interleukin 1 receptor-like 1	1.7731018	up	49.501602	47.66171	85.11361	66.08117	49.734814	0.491816437	0.07781124	0.029573917	0.85584927	0.50362253	0.09991423
7927202	0.039346345	ZNF22	zinc finger protein 22 (KOX 15)	1.76986	up	205.20158	206.91888	362.74377	385.93002	322.90555	1.148796832	-0.2502818	-0.21136777	0.6122675	0.7187309	0.4612131
8021453	0.024851004	SEC11C	SEC11 homolog C (S. cerevisiae)	1.76884	up	405.47412	382.10995	678.4805	607.96344	439.02808	0.762064551	0.08808295	-0.009518306	0.8132852	0.6718216	0.19927375
8020806	0.012464412	RNF125	ring finger protein 125	1.7646782	up	112.93723	89.340485	158.77881	199.47375	162.63075	1.586058779	0.27517462	-0.061721165	0.75768405	1.1028668	0.80215055
8074931	0.022919323	ZNF70	zinc finger protein 70	1.7550838	up	203.81377	213.2065	365.7589	359.30447	324.60114	0.957690407	0.15331364	0.20443995	1.0159799	0.9919861	0.84475154
7988426	0.0173499	SLC30A4	solute carrier family 30 (zinc transporter), member 4	1.7505823	up	366.07028	304.20447	535.99945	580.0273	409.77307	1.18994307	0.15644868	-0.10580349	0.70203143	0.81936646	0.32805762
8040473	0.013664482	RHOB	ras homolog gene family, member B	1.7437459	up	700.92084	674.32886	1170.1725	1062.9945	870.98047	0.783847182	0.029914537	-0.024894714	0.7772951	0.64554405	0.35717773
7906810	0.012256352	DUSP12	dual specificity phosphatase 12	1.7386088	up	330.49286	298.74008	512.5037	522.7224	497.11032	1.047803738	0.08897972	-0.054450512	0.7434829	0.7732366	0.6998701
8077712	0.004964114	CRELD1	cysteine-rich with EGF-like domains 1	1.7379986	up	325.37534	346.51138	599.4892	514.5421	307.24026	0.664211274	-0.18746535	-0.10214106	0.6952858	0.47512022	-0.26885667
7903619	1.86E-04	SARS	seryl-tRNA synthetase	1.7359008	up	1378.4448	1341.9222	2328.3892	2059.1638	1448.0531	0.72708119	0.0703694	0.030858994	0.8265435	0.6496865	0.14208539
8147375	0.03675287	DPY19L4	dpy-19-like 4 (C. elegans)	1.7334824	up	748.2292	701.9261	1199.7494	1220.8939	1012.7612	1.042473906	-0.029122353	-0.106498085	0.6871751	0.71409386	0.44604048
8118833	0.007053615	UHRF1BP1	UHRF1 binding protein 1	1.7311124	up	451.13586	428.19568	737.1533	689.97845	558.10767	0.847309641	0.21531391	0.13106506	0.9227645	0.831206	0.52585953

7914563	0.011836725	YARS	tyrosyl-tRNA synthetase	1.7303493	up	1786.1788	1793.0977	3123.7678	2568.4934	1594.4066	0.582710696	-0.04828008	-0.048167545	0.7428958	0.47521433	-0.21427219
7902810	0.002478396	LMO4	LIM domain only 4	1.7288637	up	165.31047	148.22134	255.61542	230.17036	165.11449	0.763068318	0.07527208	-0.07559776	0.7142264	0.5625054	0.084829964
7990080	0.002721994	LARP6	La ribonucleoprotein domain family, member 6	1.7228522	up	630.4463	638.21893	1105.2424	916.4323	764.4472	0.595716035	-0.01824824	-0.001680056	0.7831189	0.5195411	0.25943628
8009727	0.028333265	ICT1	immature colon carcinoma transcript 1	1.7201862	up	666.1768	610.7717	1035.0692	1121.7664	1015.1359	1.204331159	-0.045372646	-0.1779321	0.6046327	0.7278042	0.5822795
7939434	5.06E-04	TTC17	tetratricopeptide repeat domain 17	1.7170311	up	793.9441	874.2215	1497.4518	1219.3011	814.5809	0.553695159	-0.1812172	-0.04052512	0.739391	0.4426864	-0.1420803
7995362	7.84E-04	GPT2	glutamic pyruvate transaminase (alanine aminotransferase) 2	1.7166622	up	335.2313	324.1229	555.4082	524.40967	378.2212	0.865972762	0.11105633	0.06567097	0.84527713	0.7572009	0.2892259
7936408	0.023418786	DCLRE1A	DNA cross-link repair 1A	1.7150741	up	288.6458	238.9524	405.1857	448.97153	396.1914	1.263399872	0.16649103	-0.10284996	0.67542094	0.82989234	0.64585954
7938687	0.002850592	NUCB2	nucleobindin 2	1.7131771	up	928.4577	909.5306	1561.4971	1286.1691	836.8327	0.577696093	-0.0215861	-0.052345593	0.7243287	0.45030656	-0.17627208
8119898	0.003157177	VEGFA	vascular endothelial growth factor A	1.7114686	up	755.58466	812.2009	1383.4336	1141.8594	897.78613	0.577100191	-0.062644325	0.033656757	0.8088916	0.53189945	0.18383916
7956443	0.002205542	MARS	methionyl-tRNA synthetase	1.7002078	up	2258.5586	2323.1384	3930.9846	3401.9382	2529.6863	0.670959573	-0.029896101	0.014026324	0.7797375	0.56973076	0.1435407
8132523	0.03353111	UBE2D4	ubiquitin-conjugating enzyme E2D 4 (putative)	1.6998789	up	447.6122	470.0448	787.3663	770.8081	680.8587	0.947818852	-0.2005148	-0.13719018	0.62824184	0.6043002	0.42658648
7995552	0.010514076	CYLD	cylindromatosis (turban tumor syndrome)	1.6997728	up	210.09816	205.60329	348.21762	407.34293	345.13147	1.414581831	-0.014635404	-0.037519455	0.7278225	0.9611319	0.7194746
7928671	0.019061523	C10orf57	chromosome 10 open reading frame 57	1.6985775	up	469.05557	467.34998	791.0567	681.20044	587.4138	0.660630277	-0.17209053	-0.15389697	0.61043006	0.39484534	0.18751939
8127787	0.014947082	IBTK	inhibitor of Bruton agammaglobulinemia tyrosine kinase	1.6962317	up	480.10718	462.33362	789.64056	602.32697	444.66544	0.427712746	0.06207053	0.007452329	0.7697856	0.38594595	-0.048515003
8095303	0.007343383	LPHN3	latrophilin 3	1.6939843	up	161.65205	141.15736	241.62776	134.55766	111.68287	-0.065688004	0.20223077	0.009729703	0.7701502	-0.065491356	-0.34384075
7967072	0.014686932	COQ5	coenzyme Q5 homolog, methyltransferase (S. cerevisiae)	1.6922517	up	547.7314	488.37146	823.055	752.7629	643.73834	0.789974434	0.05979125	-0.11450227	0.6444419	0.5208092	0.29496703
8135514	0.007166177	IFRD1	interferon-related developmental regulator 1	1.6914634	up	443.1434	400.13248	681.9739	497.6005	348.39978	0.345825748	0.16135311	0.013334592	0.77160645	0.32561302	-0.18766434
8179326	0.007983442	C6orf48	chromosome 6 open reading frame 48	1.6884508	up	954.2178	947.3493	1606.9393	1891.5157	1511.8063	1.431444382	-0.019705137	-0.03460884	0.7210913	0.96283054	0.6422491
8178090	0.007954271	C6orf48	chromosome 6 open reading frame 48	1.6870383	up	955.94653	948.04083	1606.5957	1887.108	1512.0704	1.425951295	-0.018024445	-0.034543037	0.7199497	0.95823544	0.6415618
8161774	0.004885592	TRPM6	transient receptor potential cation channel, subfamily M, member 6	1.6743325	up	145.88263	143.31879	238.18703	210.13281	119.79098	0.704282276	0.02241691	-0.00502348	0.7385626	0.55332786	-0.25846544
7911718	0.001986883	LOC100129534	small nuclear ribonucleoprotein polypeptide N pseudogene	1.6731938	up	137.98839	119.94094	200.49626	150.06401	125.42377	0.373942652	0.17043893	-0.035239536	0.70736504	0.2878882	0.03125127
7927146	0.009443624	CSGALNACT2	chondroitin sulfate N-acetylgalactosaminyltransferase 2	1.6667256	up	468.68967	464.93213	773.6694	809.22314	534.0249	1.115158562	0.022857985	0.005192757	0.74220943	0.812465	0.20840995
7968344	0.022100614	ALOX5AP	arachidonate 5-lipoxygenase-activating protein	1.6589859	up	71.214096	66.102684	111.62052	78.28683	71.568016	0.267678499	0.09863424	-0.00975291	0.7205486	0.23345344	0.10367489
8174361	0.005968227	TSC22D3	TSC22 domain family, member 3	1.6569492	up	206.16425	202.75066	337.02792	279.49448	210.75304	0.571532514	-0.02513663	-0.04649989	0.6820294	0.41826725	0.012939612
7961371	0.003959347	DUSP16	dual specificity phosphatase 16	1.6510566	up	309.4222	315.9098	520.62177	494.61588	413.60953	0.872963511	-0.020578384	0.004478455	0.7278681	0.6569322	0.39678288
8018694	0.029906275	PRPSAP1	phosphoribosyl pyrophosphate synthetase-associated protein 1	1.6500921	up	331.5431	335.33243	545.2666	600.21027	586.6438	1.261718566	-0.15213823	-0.11978372	0.6027629	0.74563056	0.70905113
8051864	0.023155889	LOC728819	hCG1645220	1.6437445	up	98.52338	82.91337	137.53847	161.2342	119.85441	1.433788313	0.25920105	0.002901713	0.71988773	0.9637516	0.53273803
8143633	0.009392705	OR2A4	olfactory receptor, family 2, subfamily A, member 4	1.6433091	up	209.4356	190.82213	313.7039	254.59198	197.27538	0.518952893	0.14877796	0.00970618	0.7263101	0.42910004	0.059038322
7978801	0.007201058	MDGA2	MAM domain containing glycosylphosphatidylinositol anchor 2	1.632271	up	261.7648	226.66136	372.82678	228.93135	146.86955	0.015530281	0.17942317	-0.016815027	0.6900657	-0.004071713	-0.64497375
7978492	0.023376005	HEATR5A	HEAT repeat containing 5A	1.6309397	up	627.5982	581.19196	932.9799	786.59424	653.2994	0.58388096	0.05235513	-0.06943607	0.63626736	0.39078394	0.123078026
7920877	0.006113945	ARHGEF2	Rho/Rac guanine nucleotide exchange factor (GEF) 2	1.6300415	up	578.3471	624.79315	1021.24054	836.92944	678.6809	0.535093168	-0.14268398	-0.030748367	0.6741603	0.3848845	0.08266735
7971780	0.0168138	NEK3	NIMA (never in mitosis gene a)-related kinase 3	1.6298323	up	69.67791	78.338356	129.50104	145.7397	105.77474	1.317392653	-0.19108804	-0.013969739	0.69075376	0.8734234	0.41886362
8106098	0.001231756	MAP1B	microtubule-associated protein 1B	1.628683	up	2146.674	1957.0166	3181.0947	2200.4705	1614.2865	0.198887555	0.095742546	-0.041424114	0.6622817	0.13064289	-0.3161564
7963646	0.04896932	AAAS	achalasia, adrenocortical insufficiency, alacrimia	1.6264588	up	1416.4818	1317.1671	2095.7834	2072.1174	1908.9131	0.969605055	-0.10646979	-0.19581223	0.505922	0.49335226	0.37527528
8158671	0.024323106	ASS1	argininosuccinate synthase 1	1.6251048	up	161.60022	168.36987	278.11545	199.35968	168.81824	0.282378662	-0.023047766	0.036824863	0.7373576	0.27516556	0.032826584
8088001	0.02697911	NEK4	NIMA (never in mitosis gene a)-related kinase 4	1.6244434	up	410.87708	400.22318	638.7829	741.7591	712.17017	1.431657951	0.042998314	0.001742999	0.70168847	0.9191065	0.85899323
8059350	0.043360043	AP1S3	adaptor-related protein complex 1, sigma 3 subunit	1.6229432	up	169.08005	155.6494	253.50108	234.17659	225.45024	0.802512435	-0.031703155	-0.09425831	0.6043542	0.50960463	0.4546237
8157463	0.013612976	C9orf91	chromosome 9 open reading frame 91	1.6186119	up	431.18228	424.547	679.4196	666.00183	500.29907	0.947354992	-0.018586159	-0.052015305	0.64274186	0.6142604	0.19866149
8075182	6.74E-04	XBP1	X-box binding protein 1	1.617089	up	1154.6929	1226.3903	1981.7137	1731.9948	890.61084	0.669388106	-0.10875797	-0.019029299	0.6743698	0.4779326	-0.4852209
8178324	0.016439924	NCRNA00171	ZNRD1 antisense RNA (non-protein coding)	1.6139435	up	93.63534	86.93632	142.19182	139.12761	112.34143	0.944544706	0.065268196	-0.03134203	0.65924805	0.6325326	0.33649364
8124622	0.010265432	ZNF311	zinc finger protein 311	1.6132306	up	65.005135	59.59671	96.32996	96.57552	93.29342	1.006684952	0.21815062	0.08762661	0.7775793	0.78594637	0.7321811
8129558	0.009227568	OR2A4	olfactory receptor, family 2, subfamily A, member 4	1.6096891	up	207.27678	188.93994	304.29056	253.27466	193.2487	0.557731896	0.18527508	0.045697052	0.7324791	0.46929708	0.07946571
8103755	0.006671816	FBXO8	F-box protein 8	1.6089678	up	311.87247	277.34805	446.00626	467.41968	419.1063	1.126963401	0.08888181	-0.083274685	0.60286075	0.67507076	0.5182409
7947540	0.03262468	TRAF6	TNF receptor-associated factor 6	1.6063966	up	390.04434	349.63818	558.5208	691.96063	611.6003	1.638826868	0.037810642	-0.12939136	0.5544367	0.8718125	0.6966219
8106516	0.020119268	JMY	junction mediating and regulatory protein, p53 cofactor	1.6045481	up	442.9485	451.2544	712.9756	782.70685	551.57275	1.266433327	0.16886203	0.17458217	0.85674924	0.9897798	0.47584343
8178275	0.008939467	ZNF311	zinc finger protein 311	1.6022812	up	64.51721	59.28325	95.19534	95.62298	91.954185	1.011907967	0.20740302	0.081203304	0.7613306	0.7716549	0.7121728
8005475	0.001575798	TRIM16L	tripartite motif-containing 16-like /// tripartite motif-containing 16	1.6010133	up	1443.9999	1600.6108	2562.073	2282.4907	1623.2461	0.709211345	-0.15670872	-0.006662369	0.6723229	0.50566417	0.015154203
8032392	0.002120329	MKNK2	MAP kinase interacting serine/threonine kinase 2	1.6006588	up	702.2495	712.39667	1137.3705	1115.324	917.6662	0.948122688	0.05910142	0.07612896	0.7547948	0.7209441	0.4384006
8116494	0.048560996	ZFP62	zinc finger protein 62 homolog (mouse)	1.5942031	up	148.62605	138.5424	221.37907	225.08513	214.67705	1.044739365	0.09695021	-0.006985506	0.66585	0.7090287	0.6400037
7975787	1.47E-04	JDP2	Jun dimerization protein 2	1.5902221	up	314.33484	318.60376	506.38647	401.25912	306.0528	0.440164912	-0.058338482	-0.03040409	0.63882416	0.30175146	-0.09175523
8096919	0.018538345	ALPK1	alpha-kinase 1	1.5891827	up	212.09502	213.6793	342.9717	234.76064	170.74437	0.163051657	-0.07359743	-0.06289244	0.60539263	0.07468414	-0.40559325
7898192	0.041427694	DNAJC16	DnaJ (Hsp40) homolog, subfamily C, member 16	1.5877929	up	702.11084	715.7072	1119.621	1007.6833	844.2796	0.722867354	-0.11568197	-0.088373505	0.5786492	0.4310503	0.17713769
8176230	0.034947608	RAB39B	RAB39B, member RAS oncogene family	1.5863764	up	159.67873	143.5229	231.23729	176.79828	134.85735	0.379360559	0.14516433	-0.013412635	0.6523225	0.29227653	-0.101169586
7919305	0.039616615	PRKAB2	protein kinase, AMP-activated, beta 2 non-catalytic subunit	1.5851029	up	373.25333	382.6792	598.1473	640.3842	584.6568	1.196023913	-0.23289125	-0.15358353	0.510993	0.613595	0.48234558
8143629	0.034832224	OR2A9P	olfactory receptor, family 2, subfamily A, member 9 pseudogene	1.581697	up	294.6475	298.6777	481.2762	380.83566	294.9454	0.44993776	-0.047688484	-0.026449203	0.6350241	0.31467852	-0.047685623
7918034	0.04683903	DPH5	DPH5 homolog (S. cerevisiae)	1.5806391	up	267.71298	246.38155	389.97733	343.10287	291.04242	0.673566591	0.03820594	-0.07579724	0.5847108	0.41701636	0.17576964
8135576	0.010996575	TES	testis derived transcript (3 LIM domains)	1.5799589	up	1072.0205	942.04346	1491.9684	1243.4849	984.63184	0.548150153	0.22416496	0.038624126	0.6985111	0.44075426	0.10545635
8102877	0.046038333	CLGN	calmegin	1.5793945	up	229.70421	210.51778	336.0431	280.83963	225.5464	0.56022044	0.16882706	0.03466304	0.6940346	0.45884514	0.12806018
8152053	0.024718728	ANKRD46	ankyrin repeat domain 46	1.578538	up	189.19514	166.09514	259.32956	307.13306	331.97067	1.512723734	0.15844631	-0.028763136	0.6298258	0.8758313	0.9889396
8030128	0.013420904	PPP1R15A	protein phosphatase 1, regulatory (inhibitor) subunit 15A	1.5766745	up	1059.6239	995.23975	1554.2455	1620.6285	1436.277	1.118751909	0.11042818	0.015799204	0.672684	0.727088	0.55388993
7964089	0.01541652	PAN2	PAN2 poly(A) specific ribonuclease subunit homolog (S. cerevisiae)	1.5697855	up	826.7249	803.6407	1246.693	1015.3913	674.62787	0.477935901	0.1037089	0.052504856	0.70307225	0.39303717	-0.18863297
7919642	0.024366328	HIST2H2AB	histone cluster 2, H2ab	1.5666009	up	2137.2136	2148.7952	3344.208	3716.5515	2911.6897	1.311476922	-0.08222389	-0.08734671	0.560291	0.71134347	0.365935
7956401	3.08E-04	SHMT2	serine hydroxymethyltransferase 2 (mitochondrial)	1.5663618	up	4145.821	3953.129	6194.7944	5668.2188	4148.31	0.765096254	0.0847133	0.017630896	0.6650483	0.53757	0.08717632

8013399	0.024008296	ULK2	unc-51-like kinase 2 (C. elegans)	1.5651938	up	189.0769	168.4933	260.56027	253.19063	221.32593	0.919953486	0.047658127	-0.12337494	0.5229664	0.48220047	0.2896913
8136983	0.03958085	OR2A9P	olfactory receptor, family 2, subfamily A, member 9 pseudogene	1.563798	up	332.9329	343.52722	547.27075	432.49512	346.18222	0.436666136	-0.092357315	-0.04568863	0.59936553	0.27749157	-0.03539403
7958275	0.007633508	POLR3B	polymerase (RNA) III (DNA directed) polypeptide B	1.5635566	up	445.17227	412.62656	642.9306	539.0944	482.5674	0.549134266	0.19007206	0.07706865	0.7219	0.46958318	0.31094518
8139723	0.004411023	FKBP9L	FK506 binding protein 9-like	1.556663	up	118.38718	127.76746	198.8799	163.04128	114.20623	0.496028824	-0.17426348	-0.06582292	0.57263374	0.287398	-0.23162413
7915870	0.036720373	ATPAF1	ATP synthase mitochondrial F1 complex assembly factor 1	1.5542564	up	200.48988	187.79402	286.80838	289.1941	264.67935	1.024094687	0.012542089	-0.08717346	0.5490511	0.56142694	0.43428788
7931930	0.038582783	PRKCQ	protein kinase C, theta	1.5542355	up	103.65005	109.172356	168.1496	200.17912	177.25838	1.543082685	0.08359846	0.14595588	0.78216094	1.0422946	0.859986
7905598	0.038695376	SNAPIN	SNAP-associated protein	1.5498487	up	633.9343	561.9866	855.1076	869.00415	781.73926	1.04740892	-0.04696401	-0.20188332	0.43024412	0.4509808	0.2956562
7976766	3.53E-04	WDR25	WD repeat domain 25	1.5465051	up	185.89563	182.0321	281.31284	247.3893	206.10744	0.658306938	0.016863188	-0.012581985	0.6164296	0.43121514	0.16758044
8086482	0.006542542	ZNF445	zinc finger protein 445	1.5451906	up	675.06854	624.0408	965.1899	1182.1384	1129.6034	1.635934552	0.053244274	-0.050335567	0.57744914	0.8752921	0.808836
8165735	0.003083421	CSF2RA	colony stimulating factor 2 receptor, alpha, low-affinity (granulocyte-macrophage)	1.5447508	up	118.799706	123.06632	189.90373	154.45436	127.89704	0.469617838	-0.008430322	0.043230217	0.6706044	0.3696885	0.10099745
8084219	0.03399178	KLHL24	kelch-like 24 (Drosophila)	1.5441898	up	287.58337	252.52698	393.90585	441.1332	349.2645	1.33404815	0.2571087	0.066991486	0.69384164	0.86944073	0.5376784
7944554	0.020323345	TMEM136	transmembrane protein 136	1.5439148	up	116.300514	108.84841	170.15251	175.28845	155.57695	1.083778083	0.07408619	-0.010292213	0.6163009	0.6740839	0.50532883
8117685	0.017583216	ZKSCAN3	zinc finger with KRAB and SCAN domains 3	1.5427468	up	98.630554	89.22947	136.98218	148.19748	139.50392	1.234862063	0.26451603	0.12587674	0.75137806	0.8663809	0.7828776
8155490	0.00192449	LOC554249	hypothetical LOC554249	1.5392023	up	481.20312	505.80173	778.2713	718.11084	573.49677	0.779203013	-0.10930506	-0.03929742	0.58288544	0.46536732	0.1436599
8060949	0.005376509	ANKRD5	ankyrin repeat domain 5	1.5372932	up	146.67015	136.36429	209.53654	197.52246	153.85071	0.83581098	0.053705376	-0.053385574	0.56700677	0.48570475	0.11715063
7935627	0.002200797	GOT1	glutamic-oxaloacetic transaminase 1, soluble (aspartate aminotransferase 1)	1.5367758	up	1951.5502	1934.7926	2981.8875	2719.8914	1917.6234	0.749787627	-0.023309072	-0.026882172	0.59302455	0.4629704	-0.039273262
8176306	0.00338763	CSF2RA	colony stimulating factor 2 receptor, alpha, low-affinity (granulocyte-macrophage)	1.5321349	up	118.71347	123.15145	188.55534	154.32768	127.896065	0.476672412	-0.009845257	0.043628216	0.6591716	0.3675939	0.10080942
7898736	0.002337894	HSPC157	hypothetical LOC29092	1.5321121	up	210.54456	189.27501	290.25104	245.51732	226.145	0.556986742	0.1831379	0.02741623	0.64293814	0.4038466	0.2834638
8095139	0.039356995	SRD5A3	steroid 5 alpha-reductase 3	1.531388	up	343.02493	307.17093	463.51688	458.4738	444.39468	0.967744096	0.064870834	-0.09997082	0.51486903	0.4921271	0.4546248
8077458	0.00349041	EDEM1	ER degradation enhancer, mannosidase alpha-like 1	1.5246615	up	617.828	621.87335	951.58673	898.7111	633.4428	0.839631531	-0.016141256	-0.007282257	0.6012068	0.523976	0.020037016
8150276	0.010517878	PPAPDC1B	phosphatidic acid phosphatase type 2 domain containing 1B	1.521579	up	499.42642	490.9399	749.8833	743.94196	570.611	0.977055449	0.032548267	0.004759153	0.6103284	0.605511	0.2198035
8151457	0.013106035	HEY1	hairy/enhancer-of-split related with YRPW motif 1	1.5210949	up	78.87292	78.50875	120.604774	90.52532	76.77533	0.285456175	-0.003405889	-0.006337802	0.59877235	0.19893122	-0.044657547
8031522	0.012538771	ZNF581	zinc finger protein 581	1.5205765	up	261.91196	244.63269	369.41837	443.86816	425.79947	1.596621263	0.20325057	0.09927877	0.7038972	0.96572083	0.9097865
8151281	0.03991451	TRAM1	translocation associated membrane protein 1	1.5174199	up	1938.9447	1948.8868	2964.9355	2862.4148	1983.4419	0.899098636	0.048779488	0.05226739	0.65388775	0.61247605	0.087028824
8110399	0.01033567	B4GALT7	xylosylprotein beta 1,4-galactosyltransferase, polypeptide 7 (galactosyltransferase I)	1.515146	up	429.68066	383.928	578.44977	546.98956	551.00385	0.838268951	0.092648186	-0.03837681	0.56108	0.47055754	0.4902242
7934906	0.005518534	ACTA2	actin, alpha 2, smooth muscle, aorta	1.5142155	up	313.5538	265.46765	401.62704	379.26517	402.22916	0.835766964	0.29499802	0.050553482	0.64912397	0.5685932	0.64417535
8156633	0.007536652	KIAA1529	SUGT1-1300002K09Rik pseudogene	1.5102258	up	110.684784	109.66388	166.48662	166.35094	147.24538	0.997612224	0.030245781	0.017985502	0.61274976	0.6198104	0.44383398
8048772	9.74E-04	RHBDD1	rhomboid domain containing 1	1.5098819	up	605.09344	611.1954	921.86395	700.8754	509.8071	0.288667778	-0.030434608	-0.01686128	0.57757443	0.18165143	-0.27639866
8111457	0.02667242	RAD1	RAD1 homolog (S. pombe)	1.5089439	up	422.85144	384.86368	580.3896	684.19653	637.251	1.530911349	0.18006961	0.042675335	0.63621455	0.8824692	0.77855873
8121861	0.026779495	NCOA7	nuclear receptor coactivator 7	1.5075307	up	286.90192	278.47595	421.29385	376.0085	279.29288	0.682915447	0.13577843	0.08661413	0.67880154	0.520552	0.0958856
7987369	0.03305818	ATPBD4	ATP binding domain 4	1.5073628	up	179.47176	167.10631	255.27278	160.51291	132.06102	-0.074783532	0.13281028	0.036391895	0.6284186	-0.029936155	-0.3088239
7961829	0.019853497	BCAT1	branched chain amino-acid transaminase 1, cytosolic	1.5045705	up	2104.7278	1874.483	2823.6584	2074.886	1544.153	0.21113379	0.10452938	-0.058176357	0.5311753	0.09540272	-0.34498248
8002897	0.0032361	TMEM231	transmembrane protein 231	1.5035963	up	233.02434	208.59729	312.52844	325.23535	311.27747	1.122262767	0.113380276	-0.029391924	0.5590253	0.60386676	0.54118985
8155393	0.004356206	LOC554249	hypothetical LOC554249	1.5033603	up	266.97946	272.085	410.8436	369.17465	287.3503	0.699701856	-0.038652897	9.10E-04	0.58910084	0.43910757	0.07690557
8056491	0.002450885	SCN9A	sodium channel, voltage-gated, type IX, alpha subunit	1.5031788	up	81.60905	82.13904	123.85773	90.62582	79.69215	0.203428727	0.009299914	0.0198706	0.60788727	0.16116524	-0.027095795
7994362	0.009087144	CCDC101	coiled-coil domain containing 101	1.50276	up	502.0447	460.66653	694.41144	810.46484	771.1905	1.496495945	0.08008131	-0.04073016	0.54688454	0.7759101	0.7047415
8161451	0.004752463	LOC554249	hypothetical LOC554249	1.5017326	up	267.40588	272.0479	410.42517	370.61813	288.00314	0.712329633	-0.035800297	0.00177447	0.58840245	0.44563356	0.081113815
8009243	0.042790364	C17orf60	chromosome 17 open reading frame 60	1.5009941	up	87.725555	95.012665	145.14644	97.54429	84.01386	0.050497394	-0.083566986	0.03065904	0.6165773	0.06918796	-0.14951293
8084634	5.30E-04	DNAJB11	DnaJ (Hsp40) homolog, subfamily B, member 11	1.5000783	up	1268.5155	1311.2109	1967.1764	1968.9462	1162.2415	1.002698008	-0.046434086	0.003805796	0.58884364	0.59132165	-0.17126083
8037387	9.49E-04		---	2.2774358	down	260.91446	312.5325	138.41777	140.18652	239.6877	0.989841468	-0.3209381	-0.001963298	-1.1893736	-1.1594867	-0.4134623
8017096	0.036460076		---	2.2228432	down	144.47345	157.06801	68.10211	99.81832	162.20164	0.643501499	0.050239086	0.18469381	-0.9677124	-0.42549658	0.1860245
8043480	0.01602308		---	1.9300326	down	312.26727	352.76254	183.54497	223.12126	263.06128	0.766121863	-0.13520098	0.063915886	-0.88470936	-0.5851655	-0.35574403
7919405	0.04763453		---	1.80442	down	321.64218	368.55054	195.59859	289.81775	343.67392	0.45522927	-0.09238275	0.053483326	-0.79805183	-0.23137856	-0.09382025
7919155	0.0481038		---	1.8016558	down	320.86618	367.8326	195.48375	289.25015	342.32297	0.455949953	-0.08294741	0.06278642	-0.786537	-0.22162819	-0.08533224
8161568	0.026735732		---	1.7566613	down	308.15817	326.57648	186.27724	225.69167	253.13051	0.719068827	-0.07681719	0.029789606	-0.7830464	-0.49009815	-0.32892656
7981333	0.037372757		---	1.6405845	down	82.23382	90.84504	55.315067	70.017204	80.98941	0.586204667	-0.2985765	-0.11213335	-0.82634324	-0.48811674	-0.3041452
7904572	0.024409382		---	1.6151546	down	146.41307	141.06688	85.74535	88.93798	137.24432	0.942289557	0.16131003	0.0863266	-0.6053457	-0.6076061	0.057885487
8081818	0.011945363		---	1.6045625	down	339.29593	325.55875	202.68892	148.24821	146.3492	1.443076303	0.08267482	0.088002525	-0.59417754	-1.1069064	-1.0689793
7919600	0.049350504		---	1.5729862	down	209.28691	223.39671	142.75922	173.09644	171.80684	0.623782685	-0.17487161	-0.0905118	-0.7440179	-0.44538832	-0.4595809
7903024	0.01203444		---	1.5716616	down	67.93586	76.79313	48.730724	57.33965	70.78155	0.693222099	-0.2252744	-0.032713573	-0.68500423	-0.4512423	-0.14551036
8172270	0.038922016		---	1.5354728	down	466.22318	509.54333	335.17728	323.1923	445.58398	1.068734596	-0.13353157	0.006768545	-0.61191434	-0.67787284	-0.18978374
8089106	0.04073457		---	1.5160339	down	59.97176	65.57586	43.796062	45.906483	48.41863	0.903101902	-0.17117119	-0.037865322	-0.6381674	-0.57379436	-0.4743336
8020971	9.86E-04		---	1.7142168	up	172.86345	183.61086	313.52844	216.64131	129.00917	0.254241574	-0.082261406	0.005505085	0.78305465	0.21918695	-0.51757956
8166607	0.012559485		---	1.6147712	up	63.190777	58.338734	93.1088	54.94513	69.621254	-0.09760131	0.17080371	0.057605583	0.7489354	-0.061697006	0.31943765
8158684	0.018359117		---	1.566977	up	63.875317	70.16403	111.36735	68.39425	68.33815	-0.042952364	-0.1654021	-0.017845154	0.6301389	-0.06883192	-0.055535953
8104117	0.035469092		---	1.5375459	up	44.291668	40.138172	61.52266	63.67816	46.035503	1.100797363	0.19201772	0.07516241	0.6957919	0.7418647	0.2857194
