# Supplementary material for: Glucosamine Downregulates the IL-1β-Induced Expression of Proinflammatory Cytokine Genes in Human Synovial MH7A Cells by O-GlcNAc Modification-Dependent and -Independent Mechanisms
Source: PLoS One. 2016 Oct 24;11(10):e0165158. doi: 10.1371/journal.pone.0165158 (PMC5077170; doi:10.1371/journal.pone.0165158)
Supplement: S5 Table — (PDF) [file pone.0165158.s008.pdf]

S5 Table. GlcN-downregulated genes, whose expression was modulated by alloxan

Gene symbols (Ratio)				
EGR1 (-1.880)	SNORD29 (0.125)	MIR155 (0.198)	MYB (0.288)	SNORD80 (0.331)
SNORD30 (0.353)	JUNB(0.390)	TM4SF1 (0.403)	SNORA62 (0.419)	SNORA22 (0.464)
DUSP6 (0.468)	ZNF432 (0.478)	SNORD14E (0.498)	PDK1 (0.508)	DUSP10 (0.519)
SNORD38B (0.533)	CA9 (0.538)	PFKFB4(0.562)	SNORD44 (0.568)	HBEGF (0.570)
PLGLB1 (0.587)	SNORD75 (0.594)	FOSL2 (0.620)	SLC2A1 (0.636)	RBM14 (0.641)
SNORD36B (0.647)	ERRFI1 (0.647)	DUSP1 (0.649)	LST-3TM12 (0.666)	TNFAIP3 (0.676)
RDH10 (0.677)	ENO2 (0.680)	TIPARP (0.681)	SNORD14C (0.697)	ADM (0.699)
SNORD78 (0.705)	C6orf155 (0.705)	IL11 (0.714)	DEPDC7 (0.722)	MYOCD (0.724)
LOC100132426 (0.725)	HRH1 (0.732)	SNORD31 (0.741)	LOC642838 (0.742)	ICAM1 (0.750)
ANGPTL2 (0.753)	BCAM (0.754)	ZNF143 (0.757)	LCMT2 (0.760)	GLS (0.763)
NEDD9 (0.779)	LOC554202 (0.779)	ABCA1 (0.782)	LPXN (0.784)	AZIN1 (0.785)
LOC642838 (0.786)	SGK1 (0.788)	ANKRD13A (0.792)	DUSP5 (0.800)	ZNF841 (0.804)
SSFA2 (0.806)	SNORD3A (0.819)	KCNMB4 (0.820)	HIVEP2 (0.825)	SPINK6 (0.828)
KLF10 (0.832)	MIR221 (0.839)	SYTL2 (0.846)	DMBT1 (0.851)	BHLHE40 (0.852)
IL24 (0.866)	GRAMD3 (0.870)	ZNF114 (0.873)	GREM1 (0.884)	PCYOX1L (0.891)
GPRC5A (0.903)	FAM172B (0.912)	HS3ST3A1 (0.912)	DIO2 (0.913)	MYPN (0.920)
BDKRB1 (0.922)	IER3 (0.934)	MEST (0.936)	FLJ45248 (0.939)	LY6K (0.942)
MIR29A (0.945)	SLC20A2 (0.950)	UCA1 (0.965)	MUC1 (0.967)	RNU2-1 (0.969)
CCRL2 (0.971)	RGS4 (0.981)	ETS2 (0.982)	FGF5 (0.982)	SLC4A7 (0.987)
MIR21 (0.993)	CITED2 (0.994)	GABRE (0.995)	POP1 (0.998)	FST (0.999)

Among GlcN-downregulated 187 genes (≤1/1.5-fold, $p < 0.05$), genes whose expression was restored by alloxan are listed. To assess the effect of alloxan, the ratio (the changes of mRNA expression in the presence of both GlcN and alloxan/the changes in the presence of GlcN) was calculated and the value of <1.0 was defined as the restoration by alloxan.
