# Supplementary material for: Glucosamine Downregulates the IL-1β-Induced Expression of Proinflammatory Cytokine Genes in Human Synovial MH7A Cells by O-GlcNAc Modification-Dependent and -Independent Mechanisms
Source: PLoS One. 2016 Oct 24;11(10):e0165158. doi: 10.1371/journal.pone.0165158 (PMC5077170; doi:10.1371/journal.pone.0165158)
Supplement: S7 Table — (PDF) [file pone.0165158.s010.pdf]

S7 Table. Ct values from quantitative real-time RT-PCR

IL-1β	-	+	+	+	+
GlcN	-	-	+	+	-
Alloxan	-	-	-	+	+
TNF-α	29.43 ± 0.23	29.1 ± 0.37	30.3 ± 0.32	29.31 ± 0.33	28.13 ± 0.36
IL-8	27.43 ± 0.53	25.24 ± 0.58	25.66 ± 0.54	24.32 ± 0.62	24.86 ± 0.95
IL-6	27.43 ± 0.30	25.61±0.28	27.09±008	27.72 ± 0.20	25.84 ± 0.09
IL-24	27.82 ± 0.33	27.56 ± 0.39	28.09 ± 0.24	27.98 ± 0.08	27.88 ± 0.30
ADAMTS-1	22.91 ± 0.35	23.88 ± 0.47	23.98 ± 0.45		
ADAMTS-6	32.12 ± 0.80	32.33 ± 0.65	32.41 ± 0.48		
ADAMTS-12	22.85 ± 0.24	22.93 ± 0.23	23.39 ± 0.25		

Data are mean ± S.E. of four to eight separate experiments.
